# Instability of turing patterns in reaction-diffusion-ODE systems

**DOI:** 10.1007/s00285-016-1035-z

**Published:** 2016-06-15

**Authors:** Anna Marciniak-Czochra, Grzegorz Karch, Kanako Suzuki

**Affiliations:** 1 Institute of Applied Mathematics, Interdisciplinary Center for Scientific Computing (IWR) and BIOQUANT, University of Heidelberg, Heidelberg, 69120 Germany; 2 Instytut Matematyczny, Uniwersytet Wrocławski, pl. Grunwaldzki 2/4, Wrocław, 50-384 Poland; 3 College of Science, Ibaraki University, 2-1-1 Bunkyo, Mito, 310-8512 Japan

**Keywords:** Pattern formation, Reaction-diffusion equations, Autocatalysis, Turing instability, Unstable stationary solutions, 35B36, 35K57, 92C15, 35M33

## Abstract

The aim of this paper is to contribute to the understanding of the pattern formation phenomenon in reaction-diffusion equations coupled with ordinary differential equations. Such systems of equations arise, for example, from modeling of interactions between cellular processes such as cell growth, differentiation or transformation and diffusing signaling factors. We focus on stability analysis of solutions of a prototype model consisting of a single reaction-diffusion equation coupled to an ordinary differential equation. We show that such systems are very different from classical reaction-diffusion models. They exhibit diffusion-driven instability (turing instability) under a condition of autocatalysis of non-diffusing component. However, the same mechanism which destabilizes constant solutions of such models, destabilizes also all continuous spatially heterogeneous stationary solutions, and consequently, there exist no stable Turing patterns in such reaction-diffusion-ODE systems. We provide a rigorous result on the nonlinear instability, which involves the analysis of a continuous spectrum of a linear operator induced by the lack of diffusion in the destabilizing equation. These results are extended to discontinuous patterns for a class of nonlinearities.

## Introduction

In this paper we focus on diffusion-driven instability (DDI) in systems of equations consisting of a single reaction-diffusion equation coupled with an ordinary differential equation system. Such systems are important for systems biology applications; they arise for example in modeling of interactions between processes in cells and diffusing growth factors, such as in refs. Hock et al. (2013), Klika et al. (2012), Marciniak-Czochra (2003), Marciniak-Czochra and Kimmel (2006, 2008), Pham et al. (2011), Umulis et al. (2006). In some cases they can be obtained as homogenization limits of models describing coupling of cell-localized processes with cell-to-cell communication via diffusion in a cell assembly (Marciniak-Czochra and Ptashnyk 2008; Marciniak-Czochra 2012). Other examples are discussed e.g. in refs. Chuan et al. (2006), Evans (1975), Marciniak-Czochra et al. (2015), Mulone and Solonnikov (2009), Wang et al. (2013) and in the references therein. A detailed discussion of the DDI phenomena in the three-component systems with some diffusion coefficients equal to zero is found in the recent work (Anma et al. 2012).

*Diffusion-driven instability*, also called the *Turing instability*, is a mechanism of *de novo* pattern formation, which has been often used to explain self-organization observed in nature. DDI is a bifurcation that arises in a reaction-diffusion system, when there exists a spatially homogeneous stationary solution which is asymptotically stable with respect to spatially homogeneous perturbations but unstable to spatially heterogeneous perturbations. Models with DDI describe a process of a destabilization of stationary spatially homogeneous steady states and evolution of the system towards spatially heterogeneous steady states. DDI has inspired a vast number of mathematical models since the seminal paper of Turing (1952), providing explanations of symmetry breaking and *de novo* pattern formation, shapes of animal coat markings, and oscillating chemical reactions. We refer the reader to the monographs by Murray (2002, 2003) and to the review article (Suzuki 2011) for references on DDI in the two component reaction-diffusion systems and to the paper Satnoianu et al. (2000) in the several component systems.

However, in many applications there are components which are localized in space, which leads to systems of ordinary differential equations coupled with reaction-diffusion equations. Our main goal is to clarify in what manner such models are different from the classical Turing-type models and to demonstrate that the spatial structure of the pattern emerging via DDI cannot be determined based on linear stability analysis.

To understand the role of non-diffusive components in the pattern formation process, we focus on systems involving a single reaction-diffusion equation coupled to ODEs. It is an interesting case, since a scalar reaction-diffusion equation cannot exhibit stable spatially heterogenous patterns (Casten and Holland 1978) and hence in such models it is the ODE component that yields the patterning process. As shown in ref. Marciniak-Czochra et al. (2013), it may happen that there exist no stable stationary patterns and the emerging spatially heterogeneous structures are of a dynamical nature. In numerical simulations of such models, solutions having the form of unbounded periodic or irregular spikes have been observed (Härting and Marciniak-Czochra 2014).

Thus, the aim of this work is to investigate to which extent the results obtained in Marciniak-Czochra et al. (2013), concerning the instability of all stationary structures, apply to a general class of reaction-diffusion-ODE models with a single diffusion operator.

We focus on the following two-equation system

1.1 $$\begin{aligned}&u_t = f(u,v), \quad \,\,\text {for}\quad x\in \overline{\Omega }, \quad t>0, \end{aligned}$$

1.2 $$\begin{aligned}&v_t = \Delta v+g(u,v) \quad \text {for}\quad x\in \Omega , \quad t>0 \end{aligned}$$

in a bounded domain $\Omega \subset \mathbb {R}^N$ for $N \ge 1$, with a $C^2$-boundary $\partial \Omega$, supplemented with the Neumann boundary condition

1.3 $$\begin{aligned} \partial _{\nu }v = 0 \qquad \text {for}\quad x\in \partial \Omega , \quad t>0, \end{aligned}$$

where $\partial _\nu = \frac{\partial }{\partial \nu }$ and $\nu$ denotes the unit outer normal vector to $\partial \Omega$, and with initial data

1.4 $$\begin{aligned} u(x,0) = u_{0}(x),\qquad v(x,0) = v_{0}(x). \end{aligned}$$

The nonlinearities $f=f(u,v)$ and $g=g(u,v)$ are arbitrary $C^3$-functions. Notice that Eq. (1.2) may contain an arbitrary diffusion coefficient which, however, can be rescaled and assumed to be equal to one.

In this paper we investigate stability properties of stationary solutions of the problem (1.1)–(1.3). Our main results are Theorems 2.1 and 2.11 which assert that, under a natural assumption satisfied by a wide variety of systems, stationary solutions are unstable. We call this assumption the *autocatalysis condition* (see Theorem 2.1) following its physical motivation in the model. We show in Section 3 that this condition is satisfied *for all stationary solutions* of a wide class of systems from mathematical biology. Our results are different in continuous and discontinuous stationary solutions. In the latter case, additional assumptions on the structure of nonlinearities are required.

As a complementary result to the instability theorems, we prove Theorem 2.9 which states that each non-constant regular stationary solution intersecting (in a sense to be defined) constant steady states with the DDI property, has to satisfy the autocatalysis condition. It is a classical idea by Turing that stable patterns appear around the constant steady state in systems of reaction-diffusion equations with DDI. Mathematical results on stability of such patterns can be found, *e.g.*, in refs. Iron et al. (2004), Wei (2008), Wei and Winter (2007, 2008, 2014) and in the references therein. In the current work, combining Theorems 2.1 and 2.9, we show that *this is not the case* in the reaction-diffusion-ODE problems (1.1)–(1.4). In other words, *the same mechanism which destabilizes constant solutions of such models, destabilizes also non-constant stationary solutions*, a behavior that does not fit the usual paradigm of the reaction-diffusion-type equations. See Remark 2.10 for more details.

Mathematically, in the proof of our main result we need to consider a nonempty continuous spectrum of the linearized operator. This seems to be a novelty in the study of reaction-diffusion equations, and is caused by the absence of diffusion in one of the equations. In Section 4, we provide a rigorous proof of the nonlinear instability of steady states by using ideas from fluid dynamics equations.

The paper is organized as follows. In Section 2, we state the main results. Section 3 provides relevant mathematical biology-related examples of reaction-diffusion-ODE systems. Proofs are postponed to Sects. 4 and 5. Section 4 is devoted to showing instability of the stationary solutions under the autocatalysis condition. A proof of the instability of discontinuous solutions requires additional conditions on the model nonlinearities. In Section 5, the continuous stationary solutions are characterized and it is shown that the autocatalysis condition is satisfied in the class of reaction-diffusion-ODE problems (1.1)–(1.4) exhibiting DDI. Appendix contains additional information on the model of early carcinogenesis which was the main motivation for our research.

## Results and comments

First we formulate a condition which leads to instability of regular stationary solutions of the problem (1.1)–(1.4). Then, we show that it is the necessary condition for DDI in reaction-diffusion-ODE systems. Finally, we extend the instability results to a class of discontinuous stationary solutions satisfying additional assumptions.

### Instability of regular steady states

First, we focus on *regular stationary solutions* (*U*, *V*) of problem (1.1)–(1.3). For this, we assume that there exists a solution (not necessarily unique) of the equation $f\big (U(x),V(x)\big )=0$ that is given by the relation $U(x)=k(V(x))$ for all $x\in \Omega$ with a $C^1$-function $k=k(V)$. Then, every regular solution (*U*, *V*) of the boundary value problem

2.1 $$\begin{aligned} f(U,V)&=0 \quad \text {for}\quad x\in \overline{\Omega }, \end{aligned}$$

2.2 $$\begin{aligned} \Delta V+g(U,V)&=0\quad \text {for}\quad x\in \Omega , \end{aligned}$$

2.3 $$\begin{aligned} \partial _{\nu }V&= 0\quad \text {for}\quad x\in \partial \Omega \end{aligned}$$

satisfies the elliptic problem

2.4 $$\begin{aligned} \Delta V+h(V)&=0\quad \text {for}\quad x\in \Omega , \end{aligned}$$

2.5 $$\begin{aligned} \partial _{\nu }V&= 0 \quad \text {for}\quad x\in \partial \Omega , \end{aligned}$$

where

2.6 $$\begin{aligned} h(V)=g\big (k(V),V\big ) \qquad \text {and}\qquad U(x)=k(V(x)). \end{aligned}$$

We show that all regular stationary solutions of the problem (1.1)–(1.4) are unstable under a simple assumption on the first equation.

#### Theorem 2.1

*(Instability of regular solutions)* Let (*U*, *V*) be a regular solution of the problem (2.1)–(2.3) satisfying the following “autocatalysis condition”:

2.7 $$\begin{aligned} f_u \big (U(x_0), V(x_0)\big ) > 0 \quad \text {for some}\quad x_0 \in {\Omega }. \end{aligned}$$

Then, (*U*, *V*) is an unstable solution of the initial-boundary value problem (1.1)–(1.4).

Inequality (2.7) can be interpreted as *autocatalysis* in the dynamics of *u* at the steady state (*U*, *V*) at some point $\ x_0 \in {\Omega }$. Stability of the stationary solution is understood in the Lyapunov sense. Moreover, we prove nonlinear instability of the stationary solutions of the problem (1.1)–(1.3) and not only their linear instability, *i.e.* the instability of zero solution of the corresponding linearized problem, see Section 4 for more explanations.

Each constant solution $(\bar{u},\bar{v})\in \mathbb {R}^2$ of the problem (2.1)–(2.3) is a particular case of a regular solution. Thus, Theorem 2.1 provides a simple criterion for the diffusion-driven instability (DDI) of $(\bar{u},\bar{v})$.

#### Corollary 2.2

If a constant solution $(\bar{u}, \bar{v})$ of the problem (1.1)–(1.4) (namely, $f(\bar{u},\bar{v})=$ and $g(\bar{u},\bar{v})=0$) satisfies the inequalities

2.8 $$\begin{aligned} f_u(\bar{u},\bar{v})> 0, \qquad f_u(\bar{u},\bar{v})+g_v(\bar{u},\bar{v})< 0, \qquad \det \left( \begin{array}{cc} f_u(\bar{u},\bar{v})&{}f_v(\bar{u},\bar{v})\\ g_u(\bar{u},\bar{v})&{}g_v(\bar{u},\bar{v}) \end{array} \right) > 0, \end{aligned}$$

then it has the DDI property.

This corollary follows directly from Theorem 2.1, because the second and the third inequality in (2.8) imply that $(\bar{u}, \bar{v})$ is stable under homogeneous perturbations; see Remark 2.4 for more details.

### Sufficient conditions for autocatalysis

Next, we show that DDI in the problem (1.1)–(1.4) implies the autocatalysis condition (2.7).

We consider only a *non-degenerate* constant stationary solution $(\bar{u},\bar{v})$ of the reaction-diffusion-ODE system (1.1)–(1.3). Hence, in the remainder of this work we make the following assumption.

#### Assumption 2.3

*(Non-degeneracy of the stationary solutions)* Let all stationary solutions, i.e. vectors $(\bar{u},\bar{v})\in \mathbb {R}^2$ such that $f(\bar{u},\bar{v})=0$ and $g(\bar{u},\bar{v})=0$, satisfy

2.9 $$\begin{aligned} f_u(\bar{u},\bar{v})+g_v(\bar{u},\bar{v})\ne 0, \quad \det \left( \begin{array}{c@{\quad }c} f_u(\bar{u},\bar{v})&{}f_v(\bar{u},\bar{v})\\ g_u(\bar{u},\bar{v})&{}g_v(\bar{u},\bar{v}) \end{array} \right) \ne 0, \quad \hbox {and} \quad f_u(\bar{u},\bar{v})\ne 0. \end{aligned}$$

#### Remark 2.4

Let us note that the first two conditions in (2.9) allow us to study the asymptotic stability of $(\bar{u},\bar{v})$ treated as a solution of the corresponding system of ordinary differential equations

2.10 $$\begin{aligned} \frac{du}{dt}=f(u,v), \qquad \frac{dv}{dt}=g(u,v), \end{aligned}$$

by analyzing eigenvalues of the corresponding linearization matrix. Indeed, the conditions for linearized stability read

1. If
  2.11 $$\begin{aligned} f_u (\bar{u}, \bar{v}) + g_v (\bar{u}, \bar{v}) < 0 \qquad \hbox {and} \qquad \det \left( \begin{array}{cc} f_u(\bar{u},\bar{v})&{}f_v(\bar{u},\bar{v})\\ g_u(\bar{u},\bar{v})&{}g_v(\bar{u},\bar{v}) \end{array} \right) > 0, \end{aligned}$$
  then the Jacobi matrix
  2.12 $$\begin{aligned} \left( \begin{array}{cc} f_u(\bar{u},\bar{v})&{}f_v(\bar{u},\bar{v})\\ g_u(\bar{u},\bar{v})&{}g_v(\bar{u},\bar{v}) \end{array} \right) \end{aligned}$$
  has all eigenvalues with negative real parts, and hence $(\bar{u}, \bar{v})$ is an asymptotically stable solution of system (2.10).
2. On the other hand, if
  2.13 $$\begin{aligned} \hbox {either}\qquad f_u (\bar{u}, \bar{v}) + g_v (\bar{u}, \bar{v}) > 0 \qquad \hbox {or} \qquad \det \left( \begin{array}{cc} f_u(\bar{u},\bar{v})&{}f_v(\bar{u},\bar{v})\\ g_u(\bar{u},\bar{v})&{}g_v(\bar{u},\bar{v}) \end{array} \right) < 0, \end{aligned}$$
  then the linearization matrix (2.12) has an eigenvalue with a positive real part, and consequently, the pair $(\bar{u}, \bar{v})$ is an unstable solution of (2.10).

Now, we state a simple but fundamental property of the stationary solutions of the problem (1.1)–(1.4).

#### Proposition 2.5

Assume that (*U*, *V*) is a non-constant regular solution of the stationary problem (2.1)–(2.3). Then, there exists $x_0\in \overline{\Omega }$, such that the vector $(\bar{u},\bar{v})\equiv \big (U(x_0), V(x_0)\big )$ is a constant solution of the problem (2.1)–(2.3).

To prove Proposition 2.5, it suffices to integrate equation (2.2) over $\Omega$ and to use the Neumann boundary condition (2.3) to obtain $\int _{\Omega } g\big (U(x),V(x)\big )\;dx =0.$ Hence, there exists $x_0\in \overline{\Omega }$ such that $g\big (U(x_0),V(x_0)\big )=0$, because *U* and *V* are continuous. Thus, by Eq. (2.1), it also holds $f\big (U(x_0),V(x_0)\big )=0.$

In the case described by Proposition 2.5, we say that *a non-constant solution* (*U*, *V*) *intersects a constant solution* $(\bar{u},\bar{v})$. Now, we prove an important property of the constant solutions that are intersected by non-constant regular solutions.

#### Proposition 2.6

Let $\big (U(x),V(x)\big )$ be a regular non-constant stationary solution of problem (1.1)–(1.3) and assume that all constant stationary solutions that are intersected by (*U*, *V*) are non-degenerate, *i.e.* relations (2.9) are satisfied. Then, at least at one of those constant solutions, denoted here by $(\bar{u},\bar{v})$, the following inequality holds

2.14 $$\begin{aligned} \frac{1}{f_u(\bar{u},\bar{v})}\det \left( \begin{array}{cc} f_u(\bar{u},\bar{v})&{}f_v(\bar{u},\bar{v})\\ g_u(\bar{u},\bar{v})&{}g_v(\bar{u},\bar{v})\\ \end{array} \right) >0. \end{aligned}$$

The proof of Proposition 2.6 is based on the properties of the solutions of the elliptic Neumann problem (2.4)–(2.5) (see Theorem 5.1, below), which we prove in Sect. 5.

#### Remark 2.7

Every non-degenerate constant solution $(\bar{u},\bar{v})$ of the problem (1.1)–(1.4) satisfying inequality (2.14) is *unstable*. If both factors on the left-hand side of inequality (2.14) are positive, then, in particular, the autocatalysis condition $f_u(\bar{u},\bar{v})>0$ is satisfied. Hence, the constant solution $(\bar{u},\bar{v})$ is an unstable solution of the reaction-diffusion-ODE system (1.1)–(1.4) by Theorem 2.1. On the other hand, if both factors on the left-hand side of inequality (2.14) are negative, then, in particular, the determinant in inequality (2.14) is negative and the constant vector $(\bar{u},\bar{v})$ is an unstable solution of the corresponding kinetic system (2.10), see the alternative (2.13) in Remark 2.4.

#### Remark 2.8

It is worth to emphasize the following particular case of the phenomenon described in Remark 2.7, because we shall encounter it in our examples, further on. Suppose that the problem (1.1)–(1.4) has a non-constant regular stationary solution (*U*, *V*) intersecting *only one* constant and non-degenerate steady state $(\bar{u},\bar{v})$ which is asymptotically stable as a solution of the kinetic system (2.10). In such case, inequality (2.14) together with the second inequality in (2.11) directly imply the autocatalysis condition $f_u(\bar{u},\bar{v})>0.$ Thus, by Theorem 2.1, $(\bar{u},\bar{v})$ is an unstable solution of the reaction-diffusion-ODE problem (1.1)–(1.4), *i.e.* the constant steady state $(\bar{u},\bar{v})$ has the DDI property. Below, in Theorem 2.9, we show that the non-constant stationary solution (*U*, *V*) also satisfies the autocatalysis condition (2.7), and hence, it is unstable.

Now, we are in the position to show that the autocatalysis condition (2.7) has to be satisfied in reaction-diffusion-ODE systems (1.1)–(1.3) with non-constant regular stationary solutions which intersect constant steady states with the DDI property.

#### Theorem 2.9

Let (*U*, *V*) be a non-constant regular stationary solution of problem (1.1)–(1.4). Denote by $(\bar{u},\bar{v})$ a non-degenerate constant solution which intersects (*U*, *V*), and satisfies inequality (2.14). Assume that $(\bar{u},\bar{v})$ is an asymptotically stable solution of the kinetic system (2.10). Then, there exists $x_0\in \Omega$ such that

$$\begin{aligned} f_u\big (U(x_0),V(x_0)\big )=f_u(\bar{u},\bar{v})>0. \end{aligned}$$

The following remark emphasizes importance of the above results.

#### Remark 2.10

The instability results from Theorem 2.1 and Corollary 2.2 combined with Theorem 2.9 can be summarized in the following way. This is a classical idea that, in a system of reaction-diffusion equations with a constant solution having the DDI property, one expects stable patterns to appear around that constant steady state. Such stationary solutions are called the *Turing patterns*. For the initial-boundary value problem for a reaction-diffusion-ODE system with a single diffusion equation (1.1)–(1.3), such stationary solutions can be constructed in the case of several models of interest (see Section 3). However, the same mechanism that destabilizes constant solutions of such models, also destabilizes the non-constant solutions. In other words, *all Turing patterns in the reaction-diffusion-ODE problems* (1.1)–(1.3) *are unstable.*

### Instability of non-regular steady states

The initial-boundary value problem (1.1)–(1.4) may also have non-regular steady states in the case when the equation $f(U,V)=0$ is not uniquely solvable. Choosing different branches of solutions of the equation $f\big (U(x),V(x)\big )=0$, we obtain the relation $U(x)=k\big (V(x)\big )$ with a discontinuous, piecewise $C^1$-function *k*. Here, we recall that a pair $\big (U,V\big )\in L^\infty (\Omega )\times W^{1,2}(\Omega )$ is a *weak solution* of problem (2.1)–(2.3) if the equation $f\big (U(x),V(x)\big )=0$ is satisfied for almost all $x\in \Omega$ and if

$$\begin{aligned} -\int _\Omega \nabla V(x)\cdot \nabla \varphi (x)\;dx +\int _\Omega g\big (U(x),V(x)\big )\varphi (x)\;dx=0 \end{aligned}$$

holds for all test functions $\varphi \in W^{1,2}(\Omega )$.

In this work, we do not prove the existence of such discontinuous solutions and we refer the reader to classical works (Aronson et al. 1988; Mimura et al. 1980; Sakamoto 1990) as well as to our recent paper Marciniak-Czochra et al. (2013, Thm. 2.9) for information about how to construct such solutions to one dimensional problems using phase portrait analysis. Our goal is to formulate a counterpart of the autocatalysis condition (2.7), which leads to instability of the weak (including discontinuous) stationary solutions.

#### Theorem 2.11

Assume that (*U*, *V*) is a weak bounded solution of the problem (2.1)–(2.3) satisfying the following counterpart of the autocatalysis condition

2.15 $$\begin{aligned} \lambda _0\le f_u(U(x),V(x))\le \Lambda _0 \qquad \text {for almost all} \quad x\in \overline{\Omega }, \end{aligned}$$

for some constants $0<\lambda _0\le \Lambda _0<\infty$. Suppose, moreover, that there exists $x_0\in \Omega$ such that $f_u(U,V)$ is continuous in a neighborhood of $x_0$. Then, (*U*, *V*) is an unstable solution of the initial-boundary value problem (1.1)–(1.4).

**Fig. 1:**
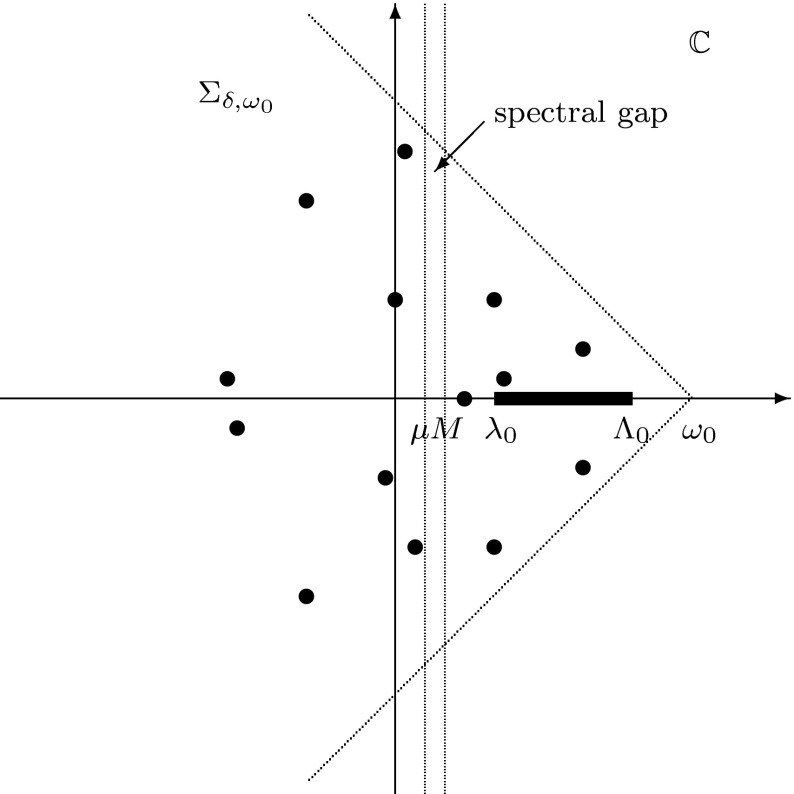
The spectrum $\sigma (\mathcal {L})$ is marked by thick dots and by the interval $[\lambda _0,\Lambda _0]$ in the sector $\Sigma _{\delta ,\omega _0}$. The spectral gap is represented by the strip $\{\lambda \in \mathbb {C}\,:\, \mu \le \mathrm{Re}\, \lambda \le M\}$ without elements of $\sigma (\mathcal {L})$

We prove Theorem 2.11 in Subsect. 4.6 by applying ideas developed for the analysis of the Euler equation and other fluid dynamics models. In that approach, it suffices to show that the spectrum of the linearized operator

$$\begin{aligned} \mathcal {L}\left( \begin{array}{c} \widetilde{u}\\ \widetilde{v}\end{array} \right) \equiv \left( \begin{array}{c} 0 \\ \Delta \widetilde{v}\end{array} \right) + \left( \begin{array}{cc} f_u(U, V)&{}f_v(U, V)\\ g_u(U, V)&{}g_v(U, V) \end{array} \right) \left( \begin{array}{c} \widetilde{u}\\ \widetilde{v}\end{array} \right) \end{aligned}$$

with the Neumann boundary condition $\partial _\nu \widetilde{v}=0,$ has so-called *spectral gap*, namely, there exists a subset of the spectrum $\sigma (\mathcal {L})$, which has a positive real part, separated from zero. Here, we prove that $\sigma (\mathcal {L})\subset \mathbb {C}$ consists of the set $\{f_u(U(x),V(x))\, :\, x\in \overline{\Omega } \}$ and of isolated eigenvalues of $\mathcal {L}$, see Section 4 and, in particular, Fig. 1 for more detail. One should emphasize that the instability of steady states from Theorems 2.1 and 2.11 is caused not by an eigenvalue with a positive real part, but rather by positive numbers from the set $\mathrm{Range}\, f_u(U,V)$ which is contained in the continuous spectrum of the operator $(\mathcal {L}, D(\mathcal {L}))$, see Theorem 4.5 below for more details.

In fact, in the case of particular nonlinearities, we do not need to assume that condition (2.15) holds true for almost all $x\in \Omega$. Indeed, if $f(0,v)=0$, one may have stationary solutions $U=U(x)$ such that $U(x)=0$ on a subset of $\Omega$ and $U(x)>0$ on a complement. Such stationary solutions can be, for example, constructed for the carcinogenezis model (3.5)–(3.7) presented below (see Marciniak-Czochra et al. (2013)), and for several other one-dimensional equations discussed in ref. Mimura et al. (1980). In the following corollary, we show instability of the discontinuous stationary solutions, under the autocatalysis condition only for $x\in \Omega$ such that $U(x)\ne 0$.

#### Corollary 2.12

*(Instability of weak solutions)* Assume that the nonlinear term in the equation (1.1) satisfies $f(0,v)=0$ for all $v\in \mathbb {R}$. Suppose that (*U*, *V*) is a weak bounded solution of the problem (2.1)–(2.3) with the following property: There exist constants $0<\lambda _0<\Lambda _0<\infty$ such that

2.16 $$\begin{aligned} \lambda _0\le f_u \big (U(x), V(x)\big ) \le \Lambda _0 \qquad \text {for almost all}\quad x\in \Omega ,\quad \text {where}\quad U(x)\ne 0. \end{aligned}$$

Moreover, suppose that there exists $x_0\in \Omega$ such that $U(x_0)\ne 0$ and the functions $U=U(x)$ and $f_u(U,V)$ are continuous in the neighborhood of $x_0$. Then, (*U*, *V*) is an unstable solution of the initial-boundary value problem (1.1)–(1.4).

#### Remark 2.13

A typical nonlinearity satisfying the assumptions of Corollary 2.12 has the form $\mathrm {f}(\mathrm {u},\mathrm {v})=\mathrm {r}(\mathrm {u},\mathrm {v})\mathrm {u}$. It can be found in the models, where the unknown variable *u* evolves according to the Malthusian law with a density dependent growth rate *r*.

We defer the proofs of Theorems 2.1 and 2.11 as well as of Corollary 2.12 to Subsection 4.6. Theorem 2.9 is somewhat independent of Theorems 2.1 and 2.11 and it is proven in Section 5.

## Model examples

In this section, our results are illustrated by applying them to some models from mathematical biology.

### Gray–Scott model

First we consider a reaction-diffusion-ODE model with nonlinearities as in the celebrated Gray-Scott system describing pattern formation in chemical reactions  (Gray and Scott 1983). The system with a non-diffusing activator takes the form

3.1 $$\begin{aligned} u_t&= - (B+k) u + u^2 v\quad \, \text {for}\quad x\in \overline{\Omega }, \; t>0, \end{aligned}$$

3.2 $$\begin{aligned} v_t&= \Delta v -u^2 v+B(1-v)\quad \text {for}\quad x\in \Omega , \; t>0, \end{aligned}$$

with the zero-flux boundary condition for *v* and with nonnegative initial conditions. The constants *B* and *k* are assumed to be positive. The system exhibits the instability phenomenon described in Sect. 2.

Here, every regular positive stationary solution (*U*, *V*) of the Neumann boundary-initial value problem for equations (3.1)–(3.2) has to satisfy the relation $U = (B+k)/ V$, hence,

3.3 $$\begin{aligned} \Delta V-B V-\frac{(B+k)^2}{V}+B&=0\quad \text {for}\quad x\in \Omega , \end{aligned}$$

3.4 $$\begin{aligned} \partial _{\nu }V&= 0 \quad \text {for}\quad x\in \partial \Omega . \end{aligned}$$

All continuous positive solutions of such boundary value problem in one dimensional case have been constructed in our recent paper Marciniak-Czochra et al. (2013, Sec. 5). A construction of discontinuous stationary solutions of the reaction-diffusion-ODE problem for (3.1)–(3.2) can be also found in Marciniak-Czochra et al. (2013, Thm. 2.9).

Instability results in Theorems 2.1 and 2.9 imply that *all stationary solutions* (constant, regular as well as discontinuous) of the reaction-diffusion-ODE problem (3.1)–(3.2) are unstable under heterogeneous perturbations. For the proof, it suffices to notice that the autocatalysis assumptions (2.7) and (2.15) are satisfied, since, for $U=(B+k)/V$, the function $f_u\big (U(x),V(x)\big )$ is independent of *x* and satisfies

$$\begin{aligned} f_u \big (U(x), V(x)\big ) = -(B+k) + 2 U(x)V(x) = B+k > 0 \qquad \text {for all} \quad x\in \Omega . \end{aligned}$$

### Model of early carcinogenesis

The main motivation for the research reported in this work has been the study of the reaction-diffusion system of three ordinary/partial differential equations modeling the diffusion-regulated growth of a cell population of the following form

3.5 $$\begin{aligned} u_t= & {} \Big (\frac{a v}{u+v} -d_c\Big ) u \quad \text {for}\ x\in \overline{\Omega }, \; t>0, \end{aligned}$$

3.6 $$\begin{aligned} v_t= & {} -d_b v +u^2 w -d v \quad \text {for}\ x\in \overline{\Omega }, \; t>0, \end{aligned}$$

3.7 $$\begin{aligned} w_t= & {} D \Delta w -d_g w -u^2 w +d v +\kappa _0 \quad \text {for}\ x\in \Omega , \; t>0, \end{aligned}$$

supplemented with zero-flux boundary conditions for the function *w* and with nonnegative initial conditions, Marciniak-Czochra et al. (2013). Here, the letters $a , d_c,d_b, d_g, d, D, \kappa _0$ denote positive constants.

The theory developed in this paper applies to a reduced two-equation version of the model (3.5)–(3.7), obtained using a quasi-steady state approximation of the dynamics of *v*. Applying the quasi-steady state approximation in Eq. (3.6) (i.e., setting $$v_t\equiv 0$$), we obtain the relation $$ v={u^2 w}/({d_b+d}), $$ which after substituting into the remaining Eqs. (3.5) and (3.7) yields the initial-boundary value problem for the following reaction-diffusion-ODE system

3.8 $$\begin{aligned}&\displaystyle u_t=\Big (\frac{a uw}{d_b+d+uw} -d_c\Big ) u \quad \text {for}\ x\in \overline{\Omega }, \; t>0, \end{aligned}$$

3.9 $$\begin{aligned}&\displaystyle w_t = D\Delta w -d_g w -\frac{d_b}{d_b+d} u^2 w +\kappa _0 \quad \text {for}\ x\in \Omega , \; t>0. \end{aligned}$$

A rigorous derivation of the two equation model (3.8)–(3.9) from the model (3.5)–(3.7) as well as other properties of the solutions to (3.8)–(3.9) are presented in Appendix A of this work. Moreover, numerical simulations suggest that the two-equation model exhibits qualitatively the same dynamics as system (3.5)–(3.7).

The autocatalysis assumptions (2.7) and (2.15) are satisfied by simple calculations, similar to those in the previous example (see Marciniak-Czochra et al. (2013) for more details). As a consequence, *all nonnegative stationary solutions* of the system (3.8)–(3.9) (regular and non-regular) are unstable due to Theorems 2.1 and 2.11. This corresponds to our results on the three-equation model (3.5)–(3.7) proved in ref. Marciniak-Czochra et al. (2013).

Stability analysis of the space homogeneous solutions of the two equation model (3.8)–(3.9) is reported in Appendix B. In particular, by Remark 2.7, constant steady states of (3.8)–(3.9) are either unstable solutions of the corresponding kinetic system or they have the DDI property.

### Model of glioma invasion

Our results can be also applied to the *“go-or-grow” model* introduced in ref. Pham et al. (2011) to investigate the dynamics of a population of glioma cells switching between a migratory and a proliferating phenotype in dependence on the local cell density. The model consists of two reaction-diffusion equations

3.10 $$\begin{aligned} u_t&=- \mu \Big (\Gamma (u+v)u-\big (1-\Gamma (u+v)\big )v\Big ) +ru\big (1-(u+v)\big ) \end{aligned}$$

3.11 $$\begin{aligned} v_t&= \Delta v +\mu \Big (\Gamma (u+v)u-\big (1-\Gamma (u+v)\big )v\Big ), \end{aligned}$$

where tumor cells are decomposed into two sub-populations: a migrating population with density *v*(*x*, *t*) and a proliferating population with density *u*(*x*, *t*) (Caution: we changed the notation from Pham et al. (2011), where $\rho _1=v$ and $\rho _2=u$). In this model, the constant $\mu >0$ is the rate at which cells change their phenotype and the constant $r\ge 0$ is the proliferation rate. The function $\Gamma =\Gamma (\rho )$ has the following explicit form

$$\begin{aligned} \Gamma (\rho )=\frac{1}{2}\big (1\pm \tanh (\alpha (\rho ^*-\rho ))\big ) \end{aligned}$$

with constant $\alpha >0$ and $\rho ^*>0$. It describes two complementary mechanisms for the phenotypic transmissions.

*Go-or-rest model.* Let us first look at a particular version of model (3.10)–(3.11) with no proliferation rate (namely $r=0$) which is called in Pham et al. (2011) as the “go-or-rest model”:

3.12 $$\begin{aligned} u_t&=- \mu \Big (\Gamma (u+v)u-\big (1-\Gamma (u+v)\big )v\Big ) \end{aligned}$$

3.13 $$\begin{aligned} v_t&= \Delta v +\mu \Big (\Gamma (u+v)u-\big (1-\Gamma (u+v)\big )v\Big ). \end{aligned}$$

One can check by a simple calculation that this system supplemented with the Neumann boundary condition for *v*(*x*, *t*) has a one parameter family of constant stationary solutions:

3.14 $$\begin{aligned} \Big (k-\Gamma (k)k, \, \Gamma (k)k\Big ) \qquad \text {for each fixed } k\in \mathbb {R}. \end{aligned}$$

These vectors are degenerate (*i.e.* they do not satisfy Assumption 2.3) because the determinant in (2.9) vanishes in this case. However, by an elementary analysis of the phase portrait of the system of the ODEs,

3.15 $$\begin{aligned} \frac{d}{dt}\bar{u}= & {} - \mu \Big (\Gamma (\bar{u}+\bar{v})\bar{u}-\big (1-\Gamma (\bar{u}+\bar{v})\big )\bar{v}\Big ),\nonumber \\ \frac{d}{dt}\bar{v}= & {} \mu \Big (\Gamma (\bar{u}+\bar{v})\bar{u}-\big (1-\Gamma (\bar{u}+\bar{v})\big )\bar{v}\Big ), \end{aligned}$$

one can show that vectors (3.14) are *stable solutions of system* (3.15). The constant steady state (3.14) satisfies the autocatalysis condition (2.7) if

3.16 $$\begin{aligned} \Gamma '(k)k+\Gamma (k)<0, \end{aligned}$$

see Pham et al. (2011, Ch. 3.1) for further discussion. Thus, by our Theorem 2.1, constant stationary solutions (3.14) are *unstable solutions* of the reaction-diffusion-ODE system (3.12)–(3.13).

System (3.12)–(3.13) has no heterogeneous stationary solutions, because the counterpart of the boundary-value problem (2.1)–(2.3) reduces in this case to the problem

$$\begin{aligned} \Delta V=0 \quad \text {in }\Omega , \qquad \partial _\nu V=0\quad \text {on }\partial \Omega , \end{aligned}$$

which has constant solutions, only.

*Go-or-grow model.* Let us now briefly sketch an analogous reasoning in the case of the more general model (3.10)–(3.11) with $r>0$. It has two constant stationary solutions (*cf.* Pham et al. (2011)):

$$\begin{aligned} (\bar{u},\bar{v})=(0,0)\quad \text {and}\quad (\bar{u},\bar{v})=\big (1-\Gamma (1),\, \Gamma (1)\big ). \end{aligned}$$

The nontrivial steady state $\big (1-\Gamma (1),\,\Gamma (1)\big )$ is a *stable solution* of the kinetic system corresponding to (3.10)–(3.11) and it satisfies the autocatalysis condition (2.7) if $(\Gamma '(1)+\Gamma (1))+(1-\Gamma (1))<0$ (see Pham et al. (2011)). In this case, by Theorem 2.1, it is an *unstable solution* of the reaction-diffusion-ODE system (3.10)–(3.11).

It is beyond the scope of this work to study positive heterogeneous stationary solutions of the go-or-grow model with $r>0$. However, if there exist regular and strictly positive stationary solutions, then under the assumption $(\Gamma '(1)+\Gamma (1))+(1-\Gamma (1))<0$, they must be unstable by Theorems 2.1 and 2.9, see also Remark 2.10. In conclusion, the structures shown in simulations of the models in ref. Pham et al. (2011) are not Turing patterns.

## Instability of the stationary solutions

### Existence of solutions

We begin our study of properties of solutions to the initial-boundary value problem (1.1)–(1.4) by recalling results on local-in-time existence and uniqueness of solutions for all bounded initial conditions.

#### Theorem 4.1

*(Local-in-time solution)* Assume that $u_0, v_0 \in L^\infty (\Omega )$. Then, there exists $T = T(\Vert u_0 \Vert _\infty ,\, \Vert v_0 \Vert _\infty ) > 0$ such that the initial-boundary value problem (1.1)–(1.4) has a unique local-in-time mild solution $u, v \in L^\infty \big ([0, T],\, L^\infty (\Omega ) \big )$.

We recall that a mild solution of problem (1.1)–(1.4) is a pair of measurable functions $u, v : [0, T] \times \overline{\Omega } \mapsto \mathbb {R}$ satisfying the following system of integral equations

4.1 $$\begin{aligned} u(x, t)&= u_0 (x) + \int _0^t f\big (u(x, s), v(x, s)\big )\, ds, \end{aligned}$$

4.2 $$\begin{aligned} v(x, t)&= e^{t\Delta }v_0 (x) + \int _0^t e^{(t-s)\Delta } g\big (u(x, s), v(x, s)\big )\, ds, \end{aligned}$$

where $e^{t\Delta }$ is the semigroup of linear operators generated by Laplacian with the Neumann boundary condition. Since our nonlinearities $f = f(u, v)$ and $g = g(u, v)$ are locally Lipschitz continuous, to construct a local-in-time unique solution of system (4.1)–(4.2), it suffices to apply the Banach fixed point theorem. Details of such a reasoning and the proof of Theorem 4.1 in a case of much more general systems of reaction-diffusion equations can be found *eg.* in Rothe (1984, Thm. 1, p. 111), see also our recent work Marciniak-Czochra et al. (2013, Ch. 3) for a construction of nonnegative solutions of particular reaction-diffusion-ODE problems.

#### Remark 4.2

If $u_0$ and $v_0$ are more regular, *i.e.* if for some $\alpha \in (0,1)$ we have $u_0 \in C^\alpha (\overline{\Omega })$, $v_0 \in C^{2+\alpha }(\overline{\Omega })$ and $\partial _\nu v_0 = 0$ on $\partial \Omega$, then the mild solution of problem (1.1)–(1.4) is smooth and satisfies $u \in C^{1, \alpha }\big ([0, T] \times \overline{\Omega } \big )$ and $v \in C^{1 + \alpha /2,\, 2 + \alpha }\left( [0, T] \times \overline{\Omega }\right)$. We refer the reader to Rothe (1984, Thm. 1, p. 112) as well as to Garroni et al. (2009) for studies of general reaction-diffusion-ODE systems in the Hölder spaces.

### Linearization of reaction-diffusion-ODE problems

Let (*U*, *V*) be a stationary solution of problem (1.1)–(1.4) — either regular as discussed in Subsection 2.1 or weak (and possibly discontinuous) as defined in Subsection 2.3. Substituting

$$\begin{aligned} u=U+\widetilde{u}\qquad \text {and}\qquad v=V+\widetilde{v}\end{aligned}$$

into (1.1)–(1.2) we obtain the initial-boundary value problem for the perturbation $(\widetilde{u},\widetilde{v})$ of the form (4.20):

4.3 $$\begin{aligned} \frac{\partial }{\partial t} \left( \begin{array}{c} \widetilde{u}\\ \widetilde{v}\end{array} \right) =\mathcal {L}\left( \begin{array}{c} \widetilde{u}\\ \widetilde{v}\end{array} \right) + \mathcal {N}\left( \begin{array}{c} \widetilde{u}\\ \widetilde{v}\end{array} \right) , \end{aligned}$$

with the Neumann boundary condition, $\partial _\nu \widetilde{v}=0,$ where the linear operator $\mathcal {L}$ and the nonlinearity $\mathcal {N}$ are defined by formulas (4.4) and (4.6), resp.

#### Lemma 4.3

Let (*U*, *V*) be a bounded (not necessarily regular) stationary solution of problem (1.1)–(1.4). We consider the following linear system

4.4 $$\begin{aligned} \left( \begin{array}{c} \widetilde{u}_t \\ \widetilde{v}_t \end{array} \right) =\mathcal {L}\left( \begin{array}{c} \widetilde{u}\\ \widetilde{v}\end{array} \right) \equiv \left( \begin{array}{c} 0 \\ \Delta \widetilde{v}\end{array} \right) + \left( \begin{array}{cc} f_u(U, V)&{}f_v(U, V)\\ g_u(U, V)&{}g_v(U, V) \end{array} \right) \left( \begin{array}{c} \widetilde{u}\\ \widetilde{v}\end{array} \right) \end{aligned}$$

with the Neumann boundary condition$\partial _\nu \widetilde{v}=0.$ Then, for every $p\in (1,\infty )$, the operator $\mathcal {L}$ with the domain $D(\mathcal {L})=L^p(\Omega )\times W_N^{2,p}(\Omega )$ generates an analytic semigroup $\{e^{t\mathcal {L}}\}_{t\ge 0}$ of linear operators on $L^p(\Omega )\times L^p(\Omega )$, which satisfies “the spectral mapping theorem”:

4.5 $$\begin{aligned} \sigma (e^{t\mathcal {L}})\setminus \{0\}=e^{t\sigma (\mathcal {L})}\qquad \text {for every} \quad t\ge 0. \end{aligned}$$

#### Proof

Here, we use the Sobolev space

$$\begin{aligned} W_N^{2,p}(\Omega )= \{u\in W^{2,p}(\Omega )\;:\; \partial _\nu u=0 \quad \text {on}\quad \partial \Omega \}. \end{aligned}$$

Notice that $\mathcal {L}$ is a bounded perturbation of the operator

$$\begin{aligned} \mathcal {L}_0 \left( \begin{array}{c} \widetilde{u}\\ \widetilde{v}\end{array} \right) \equiv \left( \begin{array}{c} 0 \\ \Delta \widetilde{v}\end{array} \right) \end{aligned}$$

with the domain $D(\mathcal {L}_0)=L^p(\Omega )\times W_N^{2,p}(\Omega )$, which generates an analytic semigroup on $L^p(\Omega )\times L^p(\Omega )$ for each $p\in (1,\infty )$. Thus, it is well-known (see *e.g.*Engel and Nagel (2000, Ch. III.1.3) and Yagi (2010, Theorems 2.15 and 2.19)) that the same property holds true for the operator $(\mathcal {L},D(\mathcal {L}))$.

The spectral mapping theorem for the semigroup $\{e^{t\mathcal {L}}\}_{t\ge 0}$ expressed by equality (4.5) holds true if the semigroup is *e.g.* eventually norm-continuous (see Engel and Nagel (2000, Ch. IV.3.10)). Since every analytic semigroup of linear operators is eventually norm-continuous, we obtain immediately relation (4.5) (cf. Engel and Nagel (2000, Ch. IV, Corollary 3.12)). $\square$

Next, we show certain elementary estimate of the nonlinearity in equation (4.3).

#### Lemma 4.4

Let (*U*, *V*) be a bounded (not necessarily regular) stationary solution of problem (1.1)–(1.4). Then, for every $p\in [1,\infty ]$, the nonlinear operator

4.6 $$\begin{aligned} \mathcal {N}\left( \begin{array}{c} \widetilde{u}\\ \widetilde{v}\end{array} \right) \equiv \left( \begin{array}{c} f(U+\widetilde{u},V+\widetilde{v})-f(U,V)\\ g(U+\widetilde{u},V+\widetilde{v})-g(U,V) \end{array} \right) - \left( \begin{array}{cc} f_u(U, V)&{}f_v(U, V)\\ g_u(U, V)&{}g_v(U, V) \end{array} \right) \left( \begin{array}{c} \widetilde{u}\\ \widetilde{v}\end{array} \right) \end{aligned}$$

satisfies

4.7 $$\begin{aligned} \left\| \mathcal {N}(\widetilde{u},\widetilde{v})\right\| _{L^p\times L^p} \le C\big (\rho , \Vert U\Vert _{L^\infty }, \Vert V\Vert _{L^\infty }\big )\Vert (\widetilde{u},\widetilde{v})\Vert _{L^\infty \times L^\infty } \Vert (\widetilde{u},\widetilde{v})\Vert _{L^p\times L^p} \end{aligned}$$

for all $\widetilde{u},\widetilde{v}\in L^\infty$ such that $\Vert \widetilde{u}\Vert _{L^\infty }<\rho$ and $\Vert \widetilde{v}\Vert _{L^\infty }<\rho$, where $\rho >0$ is an arbitrary constant. If, moreover, $U,W\in W^{1,p}(\Omega )$ then

4.8 $$\begin{aligned}&\big \Vert \nabla \mathcal {N}(\widetilde{u},\widetilde{v})\big \Vert _{L^p\times L^p}\nonumber \\&\quad \le C\big (\rho , \Vert U\Vert _{L^\infty }, \Vert V\Vert _{L^\infty }, \Vert \nabla U\Vert _{L^p}, \Vert \nabla V\Vert _{L^p}\big )\nonumber \\&\qquad \Vert (\widetilde{u},\widetilde{v})\Vert _{L^\infty \times L^\infty } \Vert (\nabla \widetilde{u},\nabla \widetilde{v})\Vert _{L^p\times L^p} \end{aligned}$$

for all $\widetilde{u},\widetilde{v}\in L^\infty$ such that $\Vert \widetilde{u}\Vert _{L^\infty }<\rho$ and $\Vert \widetilde{v}\Vert _{L^\infty }<\rho$, where $\rho >0$ is an arbitrary constant.

#### Proof

The proofs of both inequalities consist in using the Taylor formula applied to the $C^3$-nonlinearities $f=f(u,v)$ and $g=g(u,v)$ in problem (1.1)–(1.2). $\square$

### Continuous spectrum of the linear operator

Now, we are in a position to study the spectrum $\sigma (\mathcal {L})$ of the linear operator $\mathcal {L}$, given by the formula (4.4) when we linearize the reaction-diffusion-ODE problem (1.1)–(1.4) at a regular stationary solution.

#### Theorem 4.5

Assume that (*U*(*x*), *V*(*x*)) is a regular stationary solution of the problem (1.1)–(1.4) and define the constants

4.9 $$\begin{aligned} \lambda _0 = \inf _{x \in \overline{\Omega }} f_u \big (U(x), V(x)\big ) \qquad \text {and} \qquad \Lambda _0 = \sup _{x \in \overline{\Omega }} f_u \big (U(x), V(x)\big ) > 0. \end{aligned}$$

Fix $p\in (1,\infty )$. Let $\mathcal {L}$ be the linear operator defined formally by formula (4.4) with the domain $D(\mathcal {L})=L^p(\Omega )\times W_N^{2,p}(\Omega )$. Then

$$\begin{aligned}{}[\lambda _0,\Lambda _0]\subset \sigma (\mathcal {L}). \end{aligned}$$

#### Proof

We show that for each $\lambda \in [\lambda _0,\Lambda _0]$ the operator

$$\begin{aligned} \mathcal {L}-\lambda I: L^p(\Omega )\times W^{2,p}(\Omega )\rightarrow L^p(\Omega )\times L^p(\Omega ) \end{aligned}$$

defined by formula

$$\begin{aligned} (\mathcal {L}-\lambda I)(\varphi ,\psi ) = \big ( (f_u-\lambda )\varphi +f_v \psi ,\ \Delta \psi +g_u\varphi +(g_v-\lambda )\psi \big ), \end{aligned}$$

where $f_u=f_u\big (U(x),V(x)\big )$, *etc.*, cannot have a bounded inverse. Suppose, *a contrario*, that $(\mathcal {L}-\lambda I)^{-1}$ exists and is bounded. Then, for a constant $K= \Vert (\mathcal {L}-\lambda I)^{-1}\Vert$, we have

$$\begin{aligned} \Vert ( \varphi ,\psi )\Vert _{L^p(\Omega )\times W^{2,p}(\Omega )}\le K \Vert (\mathcal {L}-\lambda I)( \varphi ,\psi )\Vert _{L^p(\Omega )\times L^p(\Omega )} \end{aligned}$$

for all $( \varphi ,\psi )\in {L^p(\Omega )\times W^{2,p}(\Omega )}$ or, equivalently, using the usual norms in $L^p(\Omega )\times W^{2,p}(\Omega )$ and in $L^p(\Omega )\times L^{p}(\Omega )$:

4.10 $$\begin{aligned}&\Vert \varphi \Vert _{L^p(\Omega )} +\Vert \psi \Vert _{W^{2,p}(\Omega )}\nonumber \\&\quad \le K\big (\Vert (f_u-\lambda )\varphi +f_v\psi \Vert _{L^p(\Omega )}+ \Vert \Delta \psi +g_u\varphi +(g_v-\lambda )\psi \Vert _{L^p(\Omega )} \big ).\nonumber \\ \end{aligned}$$

A contradiction will be obtained by showing that inequality (4.10) cannot be true for all $( \varphi ,\psi )\in {L^p(\Omega )\times W^{2,p}(\Omega )}$.

To prove this claim, first we observe that, for each $\lambda \in [\lambda _0,\Lambda _0]$, there exists $x_0\in \overline{\Omega }$ such that $f_u\big (U(x_0),V(x_0)\big )-\lambda =0$. Hence, for every $\varepsilon >0$ there is a ball $B_\varepsilon \subset \Omega$ such that $\Vert f_u-\lambda \Vert _{L^\infty (B_\varepsilon )}\le \varepsilon .$

Next, for arbitrary $\psi \in C^\infty _c(\Omega )$ satisfying $\mathrm{supp}\,\psi \subset B_\varepsilon$, we choose $\varphi \in L^p(\Omega )$ such that $\mathrm{supp}\,\varphi \subset B_\varepsilon$ and in such a way that $\Delta \psi +g_u\varphi +(g_v-\lambda )\psi = \zeta$, where the function $\zeta \in L^p(\Omega )$ satisfies $\Vert \zeta \Vert _{L^p(\Omega )}\le \varepsilon \Vert \varphi \Vert _{L^p(\Omega )}$. Let us explain that such a choice of $\varphi ,\zeta \in L^p(\Omega )$ is always possible. We cut $g_u$ at the level $\varepsilon$ in the following way

$$\begin{aligned} g_u^\varepsilon = g_u^\varepsilon \big (U(x),V(x)\big ) \equiv \left\{ \begin{array}{l@{\quad }cc} g_u \big (U(x),V(x)\big ) &{}\text {if}&{} |g_u \big (U(x),V(x)\big )|>\varepsilon ,\\ \varepsilon &{}\text {if}&{} |g_u \big (U(x),V(x)\big )|\le \varepsilon . \end{array} \right. \end{aligned}$$

Thus, we obtain

$$\begin{aligned} \Delta \psi +g_u\varphi +(g_v-\lambda )\psi = \Delta \psi +g_u^\varepsilon \varphi +(g_v-\lambda )\psi + (g_u-g_u^\varepsilon )\varphi =\zeta \end{aligned}$$

for

$$\begin{aligned} \varphi = \frac{-\big (\Delta \psi +(g_v-\lambda )\psi \big )}{g_u^\varepsilon }\in L^p(\Omega ) \qquad \text {and}\qquad \zeta =(g_u-g_u^\varepsilon )\varphi \in L^p(\Omega ) \end{aligned}$$

with $$\Vert g_u-g_u^\varepsilon \Vert _{L^\infty (\Omega )}\le \varepsilon .$$

Now, noting that $\mathrm{supp}\,\varphi \subset B_\varepsilon$, we obtain the inequality

$$\begin{aligned} \Vert (f_u-\lambda ) \varphi \Vert _{L^p(\Omega )}\le \Vert (f_u-\lambda )\Vert _{L^\infty (B_\varepsilon )}\Vert \varphi \Vert _{L^p(\Omega )}\le \varepsilon \Vert \varphi \Vert _{L^p(\Omega )}. \end{aligned}$$

Thus, substituting functions $\varphi$, $\psi$, and $\zeta$ into inequality (4.10), we obtain the estimate

4.11 $$\begin{aligned}&\Vert \varphi \Vert _{L^p(\Omega )} +\Vert \psi \Vert _{W^{2,p}(\Omega )}\nonumber \\&\quad \le K\big (\Vert (f_u-\lambda )\Vert _{L^\infty (B_\varepsilon )} \Vert \varphi \Vert _{L^p(\Omega )} + \Vert f_v \psi \Vert _{L^p(\Omega )}+\Vert \zeta \Vert _{L^p(\Omega )}\big )\nonumber \\&\quad \le K\big (2\varepsilon \Vert \varphi \Vert _{L^p(\Omega )} +\Vert f_v\Vert _{L^\infty (\Omega )}\Vert \psi \Vert _{L^p(\Omega )}\big ). \end{aligned}$$

Hence, choosing $\varepsilon >0$ in such a way that $2K\varepsilon \le 1$ and compensating the term $2K\varepsilon \Vert \varphi \Vert _{L^p(\Omega )}$ on the right-hand side of inequality (4.11) by its counterpart on the left-hand side, we obtain the estimates

$$\begin{aligned} \Vert \psi \Vert _{W^{2,p}(\Omega )}\le (1-2K\varepsilon )|\varphi \Vert _{L^p(\Omega )}+ \Vert \psi \Vert _{W^{2,p}(\Omega )} \le K\Vert f_v\Vert _{L^\infty (\Omega )}\Vert \psi \Vert _{L^p(\Omega )}, \end{aligned}$$

which, obviously, cannot be true for all $\psi \in C^\infty _c(\Omega )$ such that $\mathrm{supp\,}\psi \subset B_\varepsilon$.

We have completed the proof that each $\lambda \in [\lambda _0,\Lambda _0]$ belongs to $\sigma (\mathcal {L})$. $\square$

### Eigenvalues

In the Hilbert case $D(\mathcal {L})=L^2(\Omega )\times W_N^{2,2}(\Omega )$, the remainder of the spectrum of $\big (\mathcal {L}, D(\mathcal {L})\big )$ consists of a discrete set of eigenvalues $\{\lambda _n\}_{n=1}^\infty \subset \mathbb {C}\setminus [\lambda _0,\Lambda _0]$. Here, we sketch the proof of this result, however, it does not play any role in our instability results.

As the usual practice, we analyze the corresponding resolvent equations

4.12 $$\begin{aligned} (f_u-\lambda )\varphi +f_v\psi&=F\qquad \text {in}\quad \overline{\Omega } \end{aligned}$$

4.13 $$\begin{aligned} \Delta \psi +g_u\varphi +(g_v-\lambda )\psi&=G \qquad \text {in}\quad \Omega \end{aligned}$$

4.14 $$\begin{aligned} \partial _\nu \psi&=0\qquad \text {on}\quad \partial \Omega , \end{aligned}$$

with arbitrary $F,G\in L^2(\Omega )$. Here, one should notice that for every $\lambda \in \mathbb {C}\setminus [\lambda _0,\Lambda _0]$, one can solve equation (4.12) with respect to $\varphi$. Thus, after substituting the resulting expression $\varphi =(F-f_v\psi )/(f_u-\lambda )\in L^2(\Omega )$ into (4.13), we obtain the boundary value problem

4.15 $$\begin{aligned} \Delta \psi + q ( \lambda ) \psi&= p(\lambda )\quad \text {for}\quad x\in {\Omega }, \end{aligned}$$

4.16 $$\begin{aligned} \partial _\nu \psi&= 0\quad \text {for}\quad x\in \partial \Omega , \end{aligned}$$

where

4.17 $$\begin{aligned} q(\lambda )=q(x, \lambda ) = -\frac{g_u f_v}{f_u - \lambda } + g_v - \lambda \qquad \text {and}\qquad p(\lambda )=p(x, \lambda )= G- \frac{g_u F}{f_u - \lambda }. \end{aligned}$$

For a fixed $\lambda \in \mathbb {C}\setminus [\lambda _0,\Lambda _0]$, by the Fredholm alternative, either the inhomogeneous problem (4.15)–(4.16) has a unique solution (so, $\lambda$ is not an element of $\sigma (\mathcal {L})$) or else the homogeneous boundary value problem

4.18 $$\begin{aligned} \Delta \psi + q ( \lambda ) \psi&= 0\quad \text {for}\quad x\in {\Omega }, \end{aligned}$$

4.19 $$\begin{aligned} \partial _\nu \psi&= 0\quad \text {for}\quad x\in \partial \Omega , \end{aligned}$$

has a nontrivial solution $\psi$. Hence, it suffices to consider those $\lambda \in \mathbb {C}\setminus [\lambda _0,\Lambda _0]$, for which problem (4.18)–(4.19) has nontrivial solution.

Now, we are in a position to prove that the set $\sigma (\mathcal {L})\setminus [\lambda _0,\Lambda _0]$ consists of isolated eigenvalues of $\mathcal {L}$, only. Here, it suffices to use the following general result on a family of compact operators, which we state for the reader’s convenience. The proof can be found in the Reed and Simon book Reed and Simon (1980, Thm. VI.14).

#### Theorem 4.6

*(Analytic Fredholm theorem)* Assume that *H* is a Hilbert space and denote by *L*(*H*) the Banach space of all bounded linear operators acting on *H*. For an open connected set $D\subset \mathbb {C}$, let $f:D\rightarrow L(H)$ be an analytic operator-valued function such that *f*(*z*) is compact for each $z\in D$. Then, either

- $(I-f(z))^{-1}$ exists for no $z\in D$, or
- $(I-f(z))^{-1}$ exists for all $z\in D\setminus S$, where *S* is a discrete subset of *D* (*i.e.* a set which has no limit points in *D*).

Let us rewrite problem (4.18)–(4.19) in the form

$$\begin{aligned} \psi = G\big [-(q(\lambda )+\ell )\psi \big ] \equiv R(\lambda ) \psi , \end{aligned}$$

where the operator $G=\hbox {``}{(\Delta -\ell I)^{-1}}$” supplemented with the Neumann boundary conditions is defined in the usual way. Here, $\ell \in \mathbb {R}$ is a fixed number different from each eigenvalue of Laplacian with the Neumann boundary condition.

Recall that, for each $\lambda \in \mathbb {C}\setminus [\lambda _0,\Lambda _0]$, the operator $R(\lambda ):L^2(\Omega )\rightarrow L^2(\Omega )$ is compact as the superposition of the compact operator *G* and of the continuous multiplication operator with the function $q(\lambda )+\ell \in L^\infty (\Omega )$. Moreover, the mapping $\lambda \mapsto R(\lambda )$ from the open set $\mathbb {C}\setminus [\lambda _0,\Lambda _0]$ into the Banach space of linear compact operators is analytic, which can be easily seen using the explicit form of $q(\lambda )$ in (4.17). Thus, the set $\sigma (\mathcal {L})\setminus [\lambda _0,\Lambda _0]$ consists of isolated points due to the analytic Fredholm Theorem 4.6. Here, to exclude the case (a) in Theorem 4.6, we have to show that the operator $I-R(\lambda )$ is invertible for some $\lambda \in \mathbb {C}\setminus [\lambda _0,\Lambda _0]$. This is, however, the consequence of the fact that the inhomogeneous boundary value problem (4.15)–(4.16) has a unique solution if $\lambda >0$ is chosen so large that $q(x,\lambda )<0$.

### Linearization principle

The next goal in this section is to recall that, under appropriate conditions, the linear instability of the stationary solutions of a reaction-diffusion-ODE problem implies their nonlinear instability. Such a theorem is well-known for ordinary differential equations. Furthermore, in the case of reaction-diffusion equations where the spectrum of a linearized problem is discrete, one my apply the abstract result from the book by Henry (1981, Thm.5.1.3). However, in the case of reaction-diffusion-ODE problems, the linearized operator at a stationary solution (either smooth or discontinuous) may have a non-empty continuous spectrum (*cf.* Theorem 4.5). Hence, checking the assumptions of general results from Henry (1981) does not seem to be straightforward. Therefore, here, we propose a different approach.

Let us consider a general evolution equation

4.20 $$\begin{aligned} w_t=\mathcal {L}w +\mathcal {N}(w), \qquad w(0)=w_0 \end{aligned}$$

where $\mathcal {L}$ is a generator of a $C_0$-semigroup of linear operators $\{e^{t\mathcal {L}}\}_{t\ge 0}$ on a Banach space *X* and $\mathcal {N}$ is a nonlinear operator such that $\mathcal {N}(0)=0$.

First, we recall an idea introduced by Shatah and Strauss (2000) which asserts that, under relatively strong assumption on a nonlinearity in equation (4.20), the existence of a positive part of the spectrum of the linear operator $\mathcal {L}$ is sufficient to show that the zero solution of equation (4.3) is unstable. This is the precise statement of that result.

#### Theorem 4.7

(Shatah and Strauss (2000, Thm 1)) Consider an abstract problem (4.20), where

1. the linear operator $\mathcal {L}$ generates a strongly continuous semigroup of linear operators on a Banach space *X*,
2. the intersection of the spectrum of $\mathcal {L}$ with the right half-plane $\{\lambda \in \mathbb {C}:\;: \mathrm{Re}\,\lambda >0\}$ is nonempty.
3. $\mathcal {N}:X\rightarrow X$ is continuous and there exist constants $\rho >0$, $\eta >0$, and $C>0$ such that $\Vert \mathcal {N}(w)\Vert _X \le C\Vert w\Vert _X^{1+\eta }$ for all $\Vert w\Vert _X<\rho$.

Then the zero solution of this equation is (nonlinearly) unstable.

We apply Theorem 4.7 to show an instability of regular steady states. In the case of discontinuous stationary solutions, we are unable to show that the nonlinearity in equation (4.3) satisfies the the condition (3) of Theorem 4.7. One may overcome this obstacle by assuming that the the spectrum $\sigma (\mathcal {L})$ has so-called spectral gap. This classical method has been recently used by Mulone and Solonnikov Mulone and Solonnikov (2009) to show the instability of regular stationary solutions to certain reaction-diffusion-ODE problems, however, assumptions imposed in Mulone and Solonnikov (2009) are not satisfied in our case.

The crucial idea underlying this approach is to use two Banach spaces: a “large” space *Z* where the spectrum of a linearized operator is studied and a “small” space $X\subset Z$ where an existence of solutions can be proved. More precisely, let (*X*, *Z*) be a pair of Banach spaces such that $X\subset Z$ with a dense and continuous embedding. A solution $w\equiv 0$ of the Cauchy problem (4.20) is called (*X*, *Z*)-*nonlinearly stable* if for every $\varepsilon > 0$, there exists $\delta > 0$ so that if $w(0) \in X$ and $\Vert w(0)\Vert _Z < \delta$, then

1. there exists a global in time solution of (4.20) such that $w \in C([0,\infty );X)$;
2. $$\Vert w(t)\Vert _Z < \varepsilon $$ for all $$t \in [0,\infty )$$.

An equilibrium $w\equiv 0$ that is not stable (in the above sense) is called Lyapunov unstable.

In this work, we drop the reference to the pair (*X*, *Z*). Let us also note that, under this definition of stability, a loss of the existence of a solution of (4.20) is a particular case of instability.

Now, we recall a result linking the existence of the so-called *spectral gap* to the nonlinear instability of a trivial solution to problem (4.20).

#### Theorem 4.8

We impose the two following assumptions.

1. The semigroup of linear operators $\{e^{t\mathcal {L}}\}_{t\ge 0}$ on *Z* satisfies “the spectral gap condition”, namely, we suppose that for every $t>0$ the spectrum $\sigma$ of the linear operator $e^{t\mathcal {L}}$ can be decomposed as follows: $\sigma =\sigma (e^{t\mathcal {L}})= \sigma _{-}\cup \sigma _{+}$ with $\sigma _+\ne \emptyset$, where
  $$\begin{aligned} \sigma _{-}\subset \{z\in \mathbb {C}\;:\; e^{\kappa t}<|z|<e^{\mu t}\} \quad \text {and}\quad \sigma _{+}\subset \{z\in \mathbb {C}\;:\; e^{Mt}<|z|<e^{\Lambda t}\} \end{aligned}$$
  and
  $$\begin{aligned} -\infty \le \kappa<\mu<M<\Lambda <\infty \qquad \text {for some} \quad M>0. \end{aligned}$$
2. The nonlinear term $\mathcal {N}$ satisfies the inequality
  4.21 $$\begin{aligned} \Vert \mathcal {N}(w)\Vert _Z\le C_0 \Vert w\Vert _X\Vert w\Vert _Z \qquad \text {for all}\quad w\in X \quad \text {satisfying} \quad \Vert w\Vert _X<\rho \end{aligned}$$
  for some constants $C_0>0$ and $\rho >0$.

Then, the trivial solution $w_0\equiv 0$ of the Cauchy problem (4.20) is nonlinearly unstable.

The proof of this theorem can be found in the work by Friedlander *et al.* Friedlander et al. (1997, Thm. 2.1).

#### Remark 4.9

The operator $\mathcal {L}$ considered in this work satisfies the “spectral mapping theorem”: $\sigma (e^{t\mathcal {L}})\setminus \{0\}=e^{t\sigma (\mathcal {L})}$, see Lemma 4.3. Thus, due to the relation $|e^z|=e^{\mathrm{Re}\,z}$ for every $z\in \mathbb {C}$, the spectral gap condition required in Theorem 4.8 holds true if for every $\lambda \in \sigma (\mathcal {L})$, either $\mathrm{Re}\,\lambda \in (\kappa ,\mu )$ or $\mathrm{Re}\,\lambda \in (M,\Lambda )$.

#### Remark 4.10

The authors of the reference Friedlander et al. (1997, Thm. 2.1) formulated their instability result under the spectral gap condition for a *group* of linear operators $\{e^{t\mathcal {L}}\}_{t\in \mathbb {R}}$ and in the case of a finite constant $\kappa$ (caution: in Friedlander et al. (1997), the letter $\lambda$ is used instead of $\kappa$). However, the proof of Friedlander et al. (1997, Thm. 2.1) holds true (with a minor and obvious modification) in the case of a semigroup $\{e^{t\mathcal {L}}\}_{t\ge 0}$ as well as $\kappa =-\infty$ is allowed, as stated in Theorem 4.8. This extension is important to deal with the operator $\mathcal {L}$ introduced in Lemma 4.3, which generates a semigroup of linear operators, only, and which may have an unbounded sequence of eigenvalues.

### Proofs of instability results

#### Proof of Theorem 2.1

Here, it suffices to apply Theorem 4.7 to the semi-linear equation (4.3) with the Banach space

$$\begin{aligned} X=W^{1,p}(\Omega )\times W^{1,p}(\Omega ) \qquad \text {for some}\quad p>n. \end{aligned}$$

Recall the well-known embedding $W^{1,p}(\Omega )\subset L^\infty (\Omega )$ for every $p\in (n,\infty ]$..

We refer the reader to Yagi (2010, Ch. 2) for the proof that the operator $\mathcal {L}$ discussed in Lemma 4.3 generates a semigroup of linear operators on *X*. The autocatalysis condition (2.7) combined with Theorem 4.5 imply that $\sigma (\mathcal {L})$ meets the right-hand plane of $\mathbb {C}$. Due to the embedding $X\subset L^\infty (\Omega )\times L^\infty (\Omega )$, inequalities (4.7) and (4.8) imply that the nonlinear mapping $\mathcal {N}$ in (4.6) satisfies the condition (3) of Theorem 4.7 with $\eta =1$.

Hence, the regular stationary solution (*U*, *V*) is unstable. $\square$

#### Proof of Theorem 2.11

To show an instability of non-regular stationary solution, we begin as in the proof of Theorem 2.1. First, we linearize our problem at a weak bounded stationary solution (*U*, *V*) and we notice that assumptions of Lemmas 4.3 and 4.4 are satisfied. Next, following the arguments from the proof of Theorem 4.5 we show that the number $f_u(U(x_0), V(x_0))$ belongs to $\sigma (\mathcal {L})$, where $f_u(U(x),V(x))$ is positive at $x_0$ and continuous in its neighborhood. Notice that we do not need to show that all numbers from $\mathrm{Range}\, f_u(U,V)$ are in $\sigma (\mathcal {L})$ to show the spectral gap condition required by Theorem 4.8. The reasoning from Subsection 4.4 concerning eigenvalues can be copied here without any change because $q(\lambda ,x)$ defined in (4.17) is a bounded function for every $\lambda \in \mathbb {C}\setminus [\lambda _0,\Lambda _0]$.

Now, let us show that the operator $\mathcal {L}$ has a spectral gap as required in assumption (1) of Theorem 4.8.

By Lemma 4.3, there exists a number $\omega _0\ge 0$ such that the operator $\big (\mathcal {L}-\omega _0 I, D(\mathcal {L})\big )$ generates a bounded analytic semigroup on $L^2(\Omega )\times L^2(\Omega )$; hence, this is a sectorial operator, see Engel and Nagel (2000, Ch. II, Thm. 4.6). In particular, there exists $\delta \in (0,\pi /2]$ such that $\sigma (\mathcal {L})\subset \Sigma _{\delta ,\omega _0}\equiv \{\lambda \in \mathbb {C}\;:\; |\mathrm{arg}\; (\lambda -\omega _0)|\ge \pi /2+\delta \},$ see Fig. 1. A part of the spectrum $\sigma (\mathcal {L})$ in the triangle $\Sigma _{\delta ,\omega _0}\cap \{\lambda \in \mathbb {C}\;:\, \mathrm{Re}\; \lambda >0\}$ consists of the interval $[\lambda _0,\Lambda _0]$ where $\lambda _0>0$ and of a discrete set of eigenvalues (by discussion in Subsection 4.4) with accumulation points from the interval $[\lambda _0,\Lambda _0]$, only (by Theorem 4.6.b). Thus, we can easily find infinitely many $0\le \mu <M\le \lambda _0,$ for which the spectrum $\sigma (\mathcal {L})$ can be decomposed as required in Theorem 4.8. Here, one should use the spectral mapping theorem, *i.e.* equality (4.5), and Remark 4.9.

Now, to complete the proof of an instability of not-necessarily regular stationary solutions, we apply Theorem 4.8 with $X=L^\infty (\Omega )\times L^\infty (\Omega )$ and $Z=L^2(\Omega )\times L^2(\Omega )$ for a bounded domain $\Omega \subset \mathbb {R}^N$ with a regular boundary, supplemented with the usual norms. Then, required estimate of the nonlinear mapping in (4.21) is stated in inequality (4.7) with $p=2$. $\square$

#### Proof of Corollary 2.12

Here, the analysis is similar to the case of regular stationary solutions discussed in Theorem 2.1, hence, we only emphasize the most important steps.

Let (*U*, *V*) be a weak solution of problem (2.1)–(2.3) and denote by $\mathcal {I}\subset \overline{\Omega }$ its *null* set, namely, a measurable set such that $U(x)=0$ for all $x\in \mathcal {I}$ and $U(x)\ne 0$ for all $x\in \overline{\Omega } \setminus \mathcal {I}$. For a null set $\mathcal {I}$, we define the associate $L^2$-space

$$\begin{aligned} L^2_\mathcal {I}(\Omega )=\{ v\in L^2(\Omega )\,:\, v(x)=0\quad \text {for}\quad x\in \mathcal {I}\}, \end{aligned}$$

supplemented with the usual $L^2$-scalar product, which is a Hilbert space as the closed subspace of $L^2(\Omega )$. In the same way, we define the subspace $L^\infty _\mathcal {I}(\Omega )\subset L^\infty (\Omega )$ by the equality $L^\infty _\mathcal {I}(\Omega )=\{ v\in L^\infty (\Omega )\,:\, v(x)=0\; \text {for}\; x\in \mathcal {I}\}$.

Obviously, when the measure of $\mathcal {I}$ equals zero, we have $L_\mathcal {I}^2(\Omega )=L^2(\Omega )$. The imposed assumptions imply that $\mathcal {I}$ is different from the whole interval.

Now, observe that if $u_0(x)=0$ for some $x\in \Omega$ then by equations (1.1) with the nonlinearity $f(u,v)=r(u,v)u$ we have $u(x,t)=0$ for all $t\ge 0$. Hence, the spaces

4.22 $$\begin{aligned} X_\mathcal {I}=L^\infty _\mathcal {I}(\Omega )\times L^\infty (\Omega ) \qquad \text {and}\qquad Z_\mathcal {I}=L^2_\mathcal {I}(\Omega )\times L^2(\Omega ) \end{aligned}$$

are invariant for the flow generated by problem (1.1)–(1.4) (notice that there is no “$\mathcal {I}$” in the second coordinates of $X_\mathcal {I}$ and $Z_\mathcal {I}$). The crucial part of our analysis is based on the fact that, as long as we work in the space $X_\mathcal {I}$ and $Z_\mathcal {I}$, we can linearize problem (1.1)–(1.4) at the weak solution (*U*, *V*). Moreover, for each $x\in \overline{\Omega } \setminus \mathcal {I}$, the corresponding linearized operator agrees with $\mathcal {L}$ defined in Lemma 4.3. Hence, the analysis from the proof of Theorem 2.1 can be directly adapted to discontinuous steady states in the following way.

We fix a weak stationary solution $(U_\mathcal {I}, V_\mathcal {I})$ with a null set $\mathcal {I}\subset \Omega$. The Fréchet derivative of the nonlinear mapping $\mathcal {F}: Z_\mathcal {I}\rightarrow Z_\mathcal {I}$ defined by the mappings $(U,V)\mapsto (f(U,V), g(U,V))$ at the point $(U_\mathcal {I}, V_\mathcal {I})\in Z_\mathcal {I}$ has the form

$$\begin{aligned} D\mathcal {F}(U_\mathcal {I}, V_\mathcal {I}) \left( \begin{array}{c} \varphi \\ \psi \end{array} \right) =\mathcal {A}_\mathcal {I}(x) \left( \begin{array}{c} \varphi \\ \psi \end{array} \right) , \end{aligned}$$

where

$$\begin{aligned} \mathcal {A}_\mathcal {I}(x) = \left( \begin{array}{cc} f_u(U_\mathcal {I}(x), V_\mathcal {I}(x))&{}f_v(U_\mathcal {I}(x), V_\mathcal {I}(x))\\ g_u(U_\mathcal {I}(x), V_\mathcal {I}(x))&{}g_v(U_\mathcal {I}(x), V_\mathcal {I}(x)) \end{array} \right) \end{aligned}$$

This results immediately from the definition of the Fréchet derivative.

Next, we study spectral properties of the linear operator

$$\begin{aligned} \mathcal {L}_\mathcal {I}\left( \begin{array}{c} \varphi \\ \psi \end{array} \right) = \left( \begin{array}{c@{\quad }c} 0 &{} 0\\ 0 &{} \Delta \psi \end{array} \right) +\mathcal {A}_\mathcal {I}(x) \left( \begin{array}{c} \varphi \\ \psi \end{array} \right) , \end{aligned}$$

in the Hilbert space $Z_\mathcal {I}$ (see (4.22)) with the domain $D(\mathcal {L}_\mathcal {I})= L_\mathcal {I}^2(\Omega )\times W^{2,2}(\Omega ).$ Here, the reasoning from the proof of Theorem 2.1 can be directly adapted with modifications as in the proof of Theorem 2.11.

Finally, we may study the discrete spectrum of $\mathcal {L}_\mathcal {I}$ in the same way as in Subsection 4.4 because the corresponding function $q(\lambda ,x)$ is bounded for $\lambda \in \mathbb {C}\setminus [\lambda _0,\Lambda _0]$. The proof of instability of the stationary solution $(U_\mathcal {I}, V_\mathcal {I})$ is completed by Theorem 4.8 and Lemmas 4.3-4.4. $\square$

## Constant steady states which are intersected by non-constant stationary solutions

First, we prove a certain property of stationary solutions to a general elliptic Neumann problem. This result will imply immediately Proposition 2.6.

### Theorem 5.1

Assume that $V\in C^2(\Omega )\cap C^1(\overline{\Omega })$ is a non-constant solution of the boundary value problem

5.1 $$\begin{aligned} \Delta V+h(V)=0 \quad \text {for}\ x \in \Omega \quad \text {and} \quad \partial _\nu V=0 \quad \text {for}\ x \in \partial \Omega . \end{aligned}$$

Then, there exists $x_0\in \overline{\Omega }$ and $a_0\in \mathbb {R}$ such that

5.2 $$\begin{aligned} V(x_0)=a_0, \quad h(a_0)=0 \quad \text {and}\quad h'(a_0)\ge 0. \end{aligned}$$

### Proof

First, as in the proof of Proposition 2.5, we integrate the equation in (5.1) and we use the Neumann boundary condition to obtain $$ \int _\Omega h(V(x))\, dx = 0. $$ Hence, there exists $$x_0 \in \overline{\Omega }$$ and $$a_0\in \mathbb {R}$$ such that $$ V(x_0)=a_0$$ and $$ h(a_0)=0. $$ Now, we suppose that $$h^\prime (a_0) < 0$$, and consider two cases: $$x_0 \in \Omega $$ and $$x_0 \in \partial \Omega $$, separately.

Let $x_0 \in \Omega$. Since $h(a_0) = 0$, we have

$$\begin{aligned} \Delta (V - a_0) + h(V) - h(a_0) = 0. \end{aligned}$$

Using the well-known formula

$$\begin{aligned} h(V) - h(a_0)&= \int _0^1 \frac{d}{ds}h\big (s V + (1-s)a_0\big )\, ds \\&= (V-a_0)\int _0^1 h^\prime \big (sV + (1-s)a_0\big )\, ds, \end{aligned}$$

we obtain

5.3 $$\begin{aligned} \Delta (V - a_0) + r(x, a_0) (V - a_0) = 0, \end{aligned}$$

where $r(x, a_0) = \int _0^1 h^\prime \big (sV(x) + (1-s)a_0\big )\, ds$. Observe that $r(\cdot , a_0) \in C(\Omega )$ and

$$\begin{aligned} r(x_0, a_0)&= \int _0^1 h^\prime (sV(x_0) + (1-s)a_0)\, ds \\&= \int _0^1 h^\prime (s a_0 + (1-s)a_0)\, ds \\&= h^\prime (a_0) < 0. \end{aligned}$$

Hence, there exists an open neighbourhood $\mathcal {U} \subset \Omega$ of $x_0$ such that $r (x, a_0) < 0$ for all $x \in \mathcal {U}$. Suppose that $r (x, a_0) < 0$ for all $x \in \Omega$. Multiplying both sides of equation (5.3) by $V(x)-a_0$ and integrating over $\Omega$, we obtain

$$\begin{aligned} - \int _\Omega |\nabla (V(x)-a_0)|^2\, dx + \int _\Omega r(x, a_0) (V(x) - a_0)^2\, dx = 0. \end{aligned}$$

This implies that $V(x) \equiv a_0$, which is a contradiction, because we assume that $V=V(x)$ is a non-constant solution. Therefore, there exists $x_1 \in \partial \mathcal {U} \cap \Omega$ such that $r(x_1, a_0) = 0$. It follows from equation (5.3) that $\Delta V(x_1) = 0$, and consequently, from equation (5.1) we have $h(V(x_1)) = 0$. Hence, there exists $a_1 \in \mathbb {R}$ such that $V(x_1) = a_1$ and $h(a_1) = 0$. Note that $a_1 \ne a_0$. Thus, if the equation $h(V)=0$ has only one root, the proof of Theorem 5.1 is completed.

Now, we consider two cases.

*Case I: The equation* $h(V) = 0$ *has no solution between* $a_0$ *and* $a_1$. Thus, we define the function

$$\begin{aligned} \psi (s) = V(x_0 + s (x_1 - x_0))\qquad \text {for}\ 0 \le s \le 1, \end{aligned}$$

and, without loss of generality, we can assume that $a_0< \psi (s) < a_1$ for all $0< s < 1$.

Since $$h(a_0) = 0$$ and $$h^\prime (a_0) < 0$$, we have $$h(a_0 + \theta _0) < 0$$ for small $$\theta _0 > 0$$. If we also suppose that $$h^\prime (a_1) < 0$$, then, we can find small $$\theta _1 > 0$$ such that $$h(a_1 - \theta _1) > 0$$. Noting that $$\psi (s)$$ is continuous function, we see that there exist $$s_*,\ s_{**} \in (0, 1)$$ such that $$\psi (s_*) = a_0 + \theta _0$$ and $$\psi (s_{**}) = a_1 - \theta _1$$. This implies that there exists $$ \hat{s} \in (s_*, s_{**})$$ for which $$h(V(x_0 + \hat{s} (x_1 - x_0))) = 0$$, and from the assumption for $$\psi (s)$$,

$$\begin{aligned} a_0< V(x_0 + \hat{s} (x_1 - x_0)) < a_1. \end{aligned}$$

This is a contradiction with the hypothesis that the equation $h(V) = 0$ has no roots between $a_0$ and $a_1$. Hence, $h^\prime (a_1) \ge 0$.

*Case II: The equation* $h(V) = 0$ *has a solution* $a_m$ *between* $a_0$ *and* $a_1$. It is clear that *V*(*x*) has to intersect $a_m$, too. Choosing $a_m$ the closest root of $h(V) = 0$ to $a_0$, we repeat the argument from Case I to show that $h^\prime (a_m) \ge 0$.

Next, let $x_0 \in \partial \Omega$. Following the previous reasoning and using the hypothesis $h'(a_0)<0$, we find a ball $B \subseteq \Omega$ such that $x_0 \in \partial \Omega$ and $r(x, a_0) < 0$ for all $x \in B$. Moreover, we can assume that either $V(x) > a_0$ or $V(x) < a_0$ for all $x \in B$, because, if there exists $x_1 \in B$ such that $V(x_1) = a_0$, then we can apply the same argument as in the first part of this proof to obtain $h^\prime (a_0)\ge 0$.

Let $V(x) < a_0$ for all $x \in B$, and we apply the Hopf boundary lemma to equation (5.3) in the ball *B*. If *V* is a non-constant solution satisfying $V(x) - a_0 < 0$ and $V(x_0) - a_0 = 0$, then necessarily ${\partial V(x_0)}/{\partial \nu } > 0,$ which contradicts the Neumann boundary condition satisfied by *V* at $x_0 \in \partial \Omega$.

Now, we consider the case $V(x) > a_0$ for all $x \in B$. Here, the function $U (x) = -(V(x) - a_0)$ satisfies the equation

$$\begin{aligned} -\Delta U + (- r(x, a_0))U = 0 \qquad \text {in}\ B \end{aligned}$$

where $r(x, a_0) < 0$, $U(x) < 0$ for all $x\in B$ and $U(x_0)= 0$. Hence, the Hopf boundary lemma yields a contradiction.

Thus, $h'(a_0)\ge 0$ and the proof is complete. $\square$

### Proof of Proposition 2.6

Let $\big (U(x), V(x)\big )$ be a non-constant regular stationary solution of (1.1)–(1.3) which means that $U = k(V)$ and $f\big (k(V), V \big ) =0$. Since each constant solution is non-degenerate, the equation $f(U, V) =0$ can be solved locally with respect to *U*, which implies that $k=k(V)$ is a $C^1$-function. Substituting $U = k(V)$ into equation (2.2) and denoting $h(V) = g\big (k(V), V \big )$, we obtain the following boundary value problem satisfied by $V=V(x)$:

5.4 $$\begin{aligned} \Delta V + h(V)&= 0 \quad \text {for}\ x \in \Omega , \end{aligned}$$

5.5 $$\begin{aligned} \partial _\nu V&= 0 \quad \text {for}\ x \in \partial \Omega . \end{aligned}$$

By Theorem 5.1, there exists a constant solution $\bar{v}$ of problem (5.4)–(5.5) and $x_0\in \overline{\Omega }$ such that

5.6 $$\begin{aligned} V(x_0)=\bar{v}, \quad h(\bar{v})=0, \quad \text {and}\quad h^\prime (\bar{v}) \ge 0. \end{aligned}$$

On the other hand, differentiating the function $h(v) = g\big (k(v), v\big )$ yields

5.7 $$\begin{aligned} h^\prime (v) = k^\prime (v)g_u \big (k(v), v \big ) + g_v \big (k(v), v \big ). \end{aligned}$$

Moreover, we differentiate both sides of the equation $f \big (k(v), v \big ) = 0$ with respect to *v* to obtain $k^\prime (v) f_u \big (k(v), v \big ) + f_v \big (k(v), v \big ) = 0$. Hence,

5.8 $$\begin{aligned} k^\prime (v) = - \frac{f_v \big (k(v), v \big )}{f_u \big (k(v), v \big )}. \end{aligned}$$

Finally, choosing $v=\bar{v}$ and $u=\bar{u}=k(\bar{v})$ and substituting equation (5.8) into (5.7), we obtain

$$\begin{aligned} h^\prime (\bar{v})&= - \frac{f_v \big (k(\bar{v}), \bar{v}\big )}{f_u \big (k(\bar{v}), \bar{v}\big )}g_u \big (k(\bar{v}), \bar{v}\big ) + g_v \big (k(\bar{v}), \bar{v}\big )\\&= \frac{1}{f_u (\bar{u}, \bar{v})}\big [f_u (\bar{u}, \bar{v}) g_v (\bar{u}, \bar{v}) - f_v (\bar{u}, \bar{v}) g_u (\bar{u}, \bar{v}) \big ]\\&=\frac{1}{f_u(\bar{u},\bar{v})}\det \left( \begin{array}{cc} f_u(\bar{u},\bar{v})&{}f_v(\bar{u},\bar{v})\\ g_u(\bar{u},\bar{v})&{}g_v(\bar{u},\bar{v})\\ \end{array} \right) . \end{aligned}$$

By (5.6), it holds $h'(\bar{v})\ge 0$. Moreover, since $(\bar{u}, \bar{v})$ is non-degenerate, we obtain $h'(\bar{v})>0$. This completes the proof of inequality (2.14). $\square$

## Appendix A. Model of early carcinogenesis – quasi-stationary approximation

In Appedix, we discuss in detail the model example from Subsection 3.2.

Initial-boundary value problems for reaction-diffusion-ODE systems arise in the modeling of the growth of a spatially-distributed cell population, where proliferation is controlled by endogenous or exogenous growth factors diffusing in the extracellular medium and binding to cell surface as proposed by Marciniak and Kimmel in the series of recent papers Marciniak-Czochra and Kimmel (2006, 2007, 2008). Here, we consider the following particular case of such systems, which was studied by us in Marciniak-Czochra et al. (2013)

6.1 $$\begin{aligned} \partial _t u_{\varepsilon }= & {} \Big (\frac{a v_{\varepsilon }}{u_{\varepsilon }+v_{\varepsilon }} - d_c\Big ) u_{\varepsilon } \equiv f(u_{\varepsilon },v_{\varepsilon }), \end{aligned}$$

6.2 $$\begin{aligned} \varepsilon \partial _t v_{\varepsilon }= & {} -d_b v_{\varepsilon } +u_{\varepsilon }^2 w_{\varepsilon } -d v_{\varepsilon }\equiv g(u_{\varepsilon },v_{\varepsilon },w_{\varepsilon }), \end{aligned}$$

6.3 $$\begin{aligned} \partial _t w_{\varepsilon }-D\Delta w_{\varepsilon } +d_g w_{\varepsilon }= & {} -u_{\varepsilon }^2 w_{\varepsilon } +d v_{\varepsilon } +\kappa _0 \equiv h(u_{\varepsilon },v_{\varepsilon },w_{\varepsilon }), \end{aligned}$$

on $(0,\infty )\times \Omega$, supplemented with zero-flux boundary conditions for $w_{\varepsilon }$

6.4 $$\begin{aligned} \partial _\nu w_{\varepsilon }(x,t)=0 \quad \hbox {for all} \quad t>0, \,\,x\in \partial \Omega \end{aligned}$$

and with nonnegative, smooth and bounded initial data

6.5 $$\begin{aligned} u_{\varepsilon }(x,0)=u_0(x), \quad v_{\varepsilon }(x,0)=v_0(x), \quad w_{\varepsilon }(x,0)=w_0(x). \end{aligned}$$

In equations (6.1)–(6.3), the letters $a , d_c,d_b, d_g, d, \kappa _0,D$ denote positive constants.

As it was shown in Marciniak-Czochra et al. (2013, Sec. 3), solutions of this problem are nonnegative and stay bounded on every interval [0, *T*]. Here, we study the behavior of these solutions as $\varepsilon \rightarrow 0$.

First, we notice that choosing $\varepsilon = 0$ in equation (6.2), we obtain the identity

6.6 $$\begin{aligned} v=\frac{u^2 w}{d_b+d}. \end{aligned}$$

Substituting it to the two remaining equations (6.1), (6.3) yields the system

6.7 $$\begin{aligned}&u_t=\Big (\frac{a uw}{d_b+d+uw} -d_c\Big ) u \quad \,\,\,\text {for}\ x\in \overline{\Omega }, \; t>0, \end{aligned}$$

6.8 $$\begin{aligned}&w_t = D\Delta w -d_g w -\frac{d_b}{d_b+d} u^2 w +\kappa _0 \text {for}\ x\in \Omega , \; t>0, \end{aligned}$$

6.9 $$\begin{aligned}&\partial _\nu w (x,t)=0 \quad \text {for} \ x\in \partial \Omega , \;\; t>0, \end{aligned}$$

6.10 $$\begin{aligned}&u(x,0)=u_0(x)\quad \text {and} \quad w(x,0)=w_0(x). \end{aligned}$$

Repeating our reasoning from Marciniak-Czochra et al. (2013) one can show that this new system has also a unique, global-in-time nonnegative, smooth solution for nonnegative and smooth initial data.

In this part of Appendix, we show that solutions of the quasi-stationary system (6.6)–(6.10) are good approximation of solutions of the original three-equation model (6.1)–(6.5). Our main result concerns uniform estimates for an approximation error for *u* and *w* on each finite time interval [0, *T*].

First, we show that solutions of $\varepsilon$-problem (6.1)–(6.5) are uniformly bounded with respect to small $\varepsilon >0$, locally in time.

### Lemma 6.1

For every $T>0$ there exists $C(T)>0$ such for all sufficiently small $\varepsilon >0$ (e.g. $\varepsilon \in \big (0, (d_b+d)/(2d_g)\big )$) the solution of system (6.1)–(6.5) satisfies

$$\begin{aligned} \Vert u_{\varepsilon }(t)\Vert _{L^{\infty }(\Omega )}\le C(T), \qquad \Vert v_{\varepsilon }(t)\Vert _{L^{\infty }(\Omega )}\le C(T), \qquad \Vert w_{\varepsilon }(t)\Vert _{L^{\infty }(\Omega )}\le C(T) \end{aligned}$$

for all $$t\in [0,T]$$.

It will be clear from the proof of Lemma 6.1 that the constant *C*(*T*) growths exponentially in $T>0$.

### Proof of Lemma 6.1

Since $v_\varepsilon /(u_\varepsilon +v_\varepsilon )\le 1$ for nonnegative solutions, equation (6.1) yields the inequality,

6.11 $$\begin{aligned} \Vert u_{\varepsilon }(t)\Vert _{L^{\infty }(\Omega )}\le M(t)\equiv \Vert u_0\Vert _{L^{\infty }(\Omega )} e^{(a-d_c)t}. \end{aligned}$$

Hence, we have the estimate $\Vert u_{\varepsilon }(t)\Vert _{L^{\infty }(\Omega )}\le C(T)= \Vert u_0\Vert _{L^{\infty }(\Omega )} e^{(a-d_c)T}$ for all $t\in [0,T]$.

Applying the comparison principle to the parabolic equation (6.3) with the Neumann boundary condition we obtain the estimate

6.12 $$\begin{aligned} 0 \le w_{\varepsilon } (x,t) \le \Vert w_{\varepsilon } (t)\Vert _{L^{\infty }(\Omega )} \le C_w (t), \end{aligned}$$

where the function $C_w=C_w(t)$ satisfies the Cauchy problem

6.13 $$\begin{aligned} \frac{d}{dt} C_{w}+d_g C_{w}= & {} d \Vert v_{\varepsilon }(t)\Vert _{L^{\infty }(\Omega )} +\kappa _0 \nonumber \\ C_{w}(0)= & {} \Vert w_0\Vert _{L^{\infty }(\Omega )}, \end{aligned}$$

and is given by the formula

6.14 $$\begin{aligned} C_{w}(t) = \frac{\kappa _0}{d_g} +\left( \Vert w_0\Vert _{L^{\infty }(\Omega )}- \frac{\kappa _0}{d_g} \right) e^{-d_g t} + d \int _0^t e^{-d_g(t-\tau )}\Vert v_{\varepsilon }(\tau )\Vert _{L^{\infty }(\Omega )} d\tau . \end{aligned}$$

Next, we use equation (6.2) to obtain

6.15 $$\begin{aligned} \Vert v_{\varepsilon }(t)\Vert _{L^{\infty }(\Omega )} \le \Vert v_0\Vert _{L^{\infty }(\Omega )}e^{-\frac{d_b+d}{\varepsilon }t} + \frac{1}{\varepsilon } \int _0^t M^2(\tau )\Vert w_{\varepsilon }(\tau )\Vert _{L^{\infty }(\Omega )} e^{-\frac{d_b+d}{\varepsilon }(t-\tau )} d\tau . \end{aligned}$$

Thus, using inequality (6.11) and plugging the above estimate into (6.14) yields

6.16 $$\begin{aligned} C_{w}(t)\le & {} \frac{\kappa _0}{d_g} +\left( \Vert w_0\Vert _{L^{\infty }(\Omega )}- \frac{\kappa _0}{d_g} \right) e^{-d_g t} + d \Vert v_0\Vert _{L^{\infty }(\Omega )} \int _0^t e^{-d_g(t-\tau )} e^{-\frac{d_b+d}{\varepsilon }\tau } d\tau \nonumber \\&+ \frac{ d \Vert u_0\Vert ^2_{L^{\infty }(\Omega )}}{\varepsilon } \int _0^t e^{-d_g(t-\tau )} \nonumber \\&\int _0^{\tau } e^{2(a-d_c)\xi } \Vert w_{\varepsilon }(\xi )\Vert _{L^{\infty }(\Omega )} e^{-\frac{d_b+d}{\varepsilon }(\tau -\xi )} d\xi d\tau . \end{aligned}$$

Changing the order of integration, we can simplify the last term on the right-hand side

$$\begin{aligned}&\int _0^t e^{-d_g(t-\tau )} \int _0^{\tau } e^{2(a-d_c)\xi } \Vert w_{\varepsilon }(\xi )\Vert _{L^{\infty }(\Omega )} e^{-\frac{d_b+d}{\varepsilon }(\tau -\xi )} d\xi d\tau \\&\quad = \int _0^t \frac{\varepsilon \Vert w_{\varepsilon }(\xi )\Vert _{L^{\infty }(\Omega )} }{d_b+d- \varepsilon d_g}e^{-d_g(t-\xi )}e^{2(a-d_c)\xi } d\xi \\&\qquad - \int _0^t \frac{\varepsilon \Vert w_{\varepsilon }(\xi )\Vert _{L^{\infty }(\Omega )} }{d_b+d- \varepsilon d_g}e^{2(a-d_c)\xi }e^{\frac{d_b+d}{\varepsilon }(\xi -t)} d\xi \end{aligned}$$

hence, for $0<\varepsilon < \frac{d_b+d}{d_g}$, using inequalities (6.12) we obtain

6.17 $$\begin{aligned} \Vert w_{\varepsilon } (t)\Vert _{L^{\infty }(\Omega )} \le C + \frac{d \Vert u_0\Vert ^2_{L^{\infty }(\Omega )} }{d_b+d- \varepsilon d_g} \int _0^t e^{2(a-d_c)\xi } \Vert w_{\varepsilon }(\xi )\Vert _{L^{\infty }(\Omega )}e^{-d_g(t-\xi )}d\xi , \end{aligned}$$

where $C=C( \Vert w_0\Vert _{L^{\infty }(\Omega )}, \Vert v_0\Vert _{L^{\infty }(\Omega )})$ is independent of *T* and of $\varepsilon$. Finally, the Gronwall inequality applied to (6.17) implies the estimate

6.18 $$\begin{aligned} \Vert w_{\varepsilon } (t)\Vert _{L^{\infty }(\Omega )} \le C e^{C_1(T)}\qquad \hbox {for all} \quad 0\le t \le T \end{aligned}$$

and for all sufficiently small $\varepsilon >0$ (*e.g.* $\varepsilon \in \big (0, (d_b+d)/(2d_g)\big )$), where positive constants *C* and $C_1(T)$ are independent of $\varepsilon$.

Finally, estimate (6.18) applied to inequality (6.15) implies an analogous bound for $v_{\varepsilon }$. $\square$

### Theorem 6.2

Let $(u_{\varepsilon },v_{\varepsilon },w_{\varepsilon })$ be a solution of system (6.1)- (6.5) with sufficiently small $\varepsilon >0$ (e.g. $\varepsilon \in \big (0, (d_b+d)/(2d_g)\big )$) and (*u*, *w*) be a solution of the corresponding system (6.6)–(6.10). For each $T>0$, there exists a constant $C(T)>0$ independent of $\varepsilon$ such that

6.19 $$\begin{aligned} \max _{ t\in [0,T]}\Vert u_{\varepsilon }(t)-u(t)\Vert _{L^\infty (\Omega )}\le & {} C(T) \varepsilon , \end{aligned}$$

6.20 $$\begin{aligned} \max _{ t\in [0,T]}\Vert w_{\varepsilon }(t)-w(t)\Vert _{L^\infty (\Omega )}\le & {} C(T) \varepsilon . \end{aligned}$$

Additionally, we also have

6.21 $$\begin{aligned} \int _0^T\Vert v_{\varepsilon }(t)-v(t)\Vert _{L^\infty (\Omega )} \le C(T) \varepsilon , \end{aligned}$$

where *v* is given by equation (6.6).

### Proof

Letting $\alpha =u_{\varepsilon }-u$, $\beta =v_{\varepsilon }-v$ and $\delta =w_{\varepsilon }-w$, we obtain by the Taylor expansion the following system

6.22 $$\begin{aligned}&\partial _t{\alpha }=f(u_{\varepsilon },v_{\varepsilon }) -f(u,v)= -d_c\alpha + f_1\alpha + f_2\beta , \end{aligned}$$

6.23 $$\begin{aligned}&\varepsilon \partial _t {\beta } = g(u_{\varepsilon },v_{\varepsilon },w_{\varepsilon }) -g(u,v,w)- \varepsilon \partial _t v =-(d+d_b)\beta +g_1\alpha + g_2 \delta -\varepsilon \partial _t v, \end{aligned}$$

6.24 $$\begin{aligned}&\partial _t {\delta } -D\Delta {\delta }_{xx} +(d_g+g_2) {\delta }= h_1 \alpha + d\beta , \end{aligned}$$

supplemented with the initial conditions

$$\begin{aligned} \alpha (x,0)=0, \qquad \delta (x,0)=0, \qquad \beta (x,0)=v_0(x)-\widetilde{v}_0(x) \end{aligned}$$

with $\widetilde{v}_0$ obtained from $u_0$ and $w_0$ via formula (6.6), and with the Neumann boundary condition for $\delta (x,t)$. In equations (6.22)–(6.24) the following coefficients

$$\begin{aligned} f_1=&\frac{\partial f}{\partial u}+d_c=\frac{av^2}{(u+v)^2},\qquad f_2=\frac{\partial f}{\partial v}=\frac{au^2}{(u+v)^2},\\ g_1=&\frac{\partial g}{\partial u}=2uw,\qquad g_2=\frac{\partial g}{\partial w}=-\frac{\partial h}{\partial w}=u^2,\qquad h_1=\frac{\partial h}{\partial u}=-2uw \end{aligned}$$

are calculated in certain intermediate points and are bounded independently of $\varepsilon$ due to Lemma 6.1.

The proof is divided into three steps.

*Step 1:* First, applying the comparison principle to the parabolic equation (6.24) with the Neumann boundary condition and with the zero initial datum we obtain the estimate

$$\begin{aligned} \Vert \delta (\cdot ,t)\Vert _{L^{\infty }(\Omega )}\le C_\delta (t) \qquad \text {for every}\quad t\in [0,T], \end{aligned}$$

where $C_\delta$ is a solution of the Cauchy problem

$$\begin{aligned}&C_{\delta }(0)=0,\\&\frac{d}{dt} C_{\delta }+d_g C_{\delta } = \Vert h_1\Vert _{L^\infty (\Omega \times [0,T])}\Vert \alpha (t)\Vert _{L^{\infty }(\Omega )} + d \Vert \beta (t)\Vert _{L^{\infty }(\Omega )}. \end{aligned}$$

Since

$$\begin{aligned} C_{\delta }(t) =\int _0^t e^{-d_g(t-\tau )} \big (\Vert h_1\Vert _{L^{\infty }(\Omega \times [0,T])}\Vert \alpha (\tau )\Vert _{L^{\infty }(\Omega )} + d \Vert \beta (\tau )\Vert _{L^{\infty }(\Omega )}\big )d\tau \end{aligned}$$

using the Young inequality for a convolution and the estimate $e^{-d_g(t-\tau )}\le 1$, we obtain

6.25 $$\begin{aligned} \Vert \delta (\cdot ,t)\Vert _{L^{\infty }(\Omega )}\le & {} \Vert C_{\delta }\Vert _{L^{\infty }(0,t)} \nonumber \\\le & {} \Vert h_1\Vert _{L^{\infty }(\Omega \times [0,T])} \Vert \alpha \Vert _{L^1(0,t;L^{\infty }(\Omega ))} + d \Vert \beta \Vert _{L^1(0,t;L^{\infty }(\Omega )).} \end{aligned}$$

*Step 2:* Next, we estimate the solution $\beta =\beta (x,t)$ of equation (6.23). First, note that

$$\begin{aligned} \left\| e^{-(d+d_b)\frac{\tau }{\varepsilon }}\right\| _{L^{1}(0,t)} \le \frac{\varepsilon }{d+d_b} \qquad \text {for all } \quad t>0. \end{aligned}$$

The solution of equation (6.23) satisfies the formula

$$\begin{aligned} \beta (x,t) = \beta (x,0) e^{-\frac{d+d_b}{\varepsilon }t} + \int _0^t e^{-\frac{d+d_b}{\varepsilon }(t-\tau )}\left( -\partial _{\tau }v + \frac{1}{\varepsilon } (g_1\alpha + g_2 \delta )\right) d{\tau }. \end{aligned}$$

Consequently, the Young inequality yields

6.26 $$\begin{aligned} \Vert {\beta }\Vert _{L^1(0,t;L^{\infty }(\Omega ))}\le & {} \left( \Vert {\beta (0)}\Vert _{L^{\infty }(\Omega )} + \Vert \partial _{\tau } v\Vert _{L^1(0,t;L^{\infty }(\Omega ))}\right) C \varepsilon \nonumber \\&+ C \Vert g_1\Vert _{L^{\infty }(\Omega \times [0,T])} \Vert \alpha \Vert _{L^1(0,t;L^{\infty }(\Omega ))}\nonumber \\&+ C \Vert g_2\Vert _{L^{\infty }(\Omega \times [0,T])} \Vert \delta \Vert _{L^1(0,t;L^{\infty }(\Omega ))}\nonumber \\\le & {} C\varepsilon + C\left( \Vert g_1\Vert _{L^{\infty }(\Omega \times [0,T])} \right. \nonumber \\&\left. + t\Vert h_1\Vert _{L^{\infty }(\Omega \times [0,T])} \Vert g_2\Vert _{L^{\infty }(\Omega \times [0,T])} \right) \Vert {\alpha }\Vert _{L^1(0,t;L^{\infty }(\Omega ))}\nonumber \\&+Ct d \Vert g_2\Vert _{L^{\infty }(\Omega \times [0,T])} \Vert {\beta }\Vert _{L^1(0,t;L^{\infty }(\Omega ))}, \end{aligned}$$

where the last inequality results from (6.25). Here, in the first inequality of (6.26), the function *v*(*x*, *t*) is given by formula (6.6). Hence, the quantity $\Vert \partial _{\tau } v\Vert _{L^1(0,t;L^{\infty }(\Omega ))}$ is finite (and obviously independent of $\varepsilon$) for smooth solutions by equations (6.7) and (6.8).

*Step 3:* Finally, we estimate the solution $\alpha =\alpha (x,t)$ of equation (6.22). Note that $\alpha (x,0)=0$ and

$$\begin{aligned} \alpha (t,x) = \int _0^t \left( f_2(\tau )\beta (\tau ) e^{-d_c (t-\tau ) + \int _{\tau }^t f_1(\xi )d{\xi }}\right) d\tau . \end{aligned}$$

Thus, using the Young inequality again we obtain the estimate

6.27 $$\begin{aligned} \Vert {\alpha }\Vert _{L^{\infty }((0,t)\times \Omega )} \le C e^{(a-d_c)T} \Vert f_2\Vert _{L^{\infty }(\Omega )} \Vert \beta \Vert _{L^1(0,t;L^{\infty }(\Omega ))} \end{aligned}$$

as well as

6.28 $$\begin{aligned} \Vert {\alpha }\Vert _{L^1(0,t;L^{\infty }(\Omega ))} \le Ct \Vert \beta \Vert _{L^1(0,t;L^{\infty }(\Omega ))}. \end{aligned}$$

Inserting inequality (6.28) into (6.26) leads to

6.29 $$\begin{aligned} \Vert {\beta }\Vert _{L^1(0,t;L^{\infty }(\Omega ))} \le C\varepsilon + C t \Vert {\beta }\Vert _{L^1(0,t;L^{\infty }(\Omega ))}. \end{aligned}$$

For $t\le t_0=\frac{1}{2C}$ we conclude that

6.30 $$\begin{aligned} \Vert {\beta }\Vert _{L^1(0,t;L^{\infty }(\Omega ))} \le C\varepsilon \qquad \text {for all}\quad t\le t_0. \end{aligned}$$

Since every $t\in (t_0,T]$ can be reached after a finite number of steps, estimate (6.30) holds for every $t\in [0,T]$. Furthermore, inequality (6.30) applied in (6.25) and (6.27) completes the proof of estimates (6.19)–(6.21). $\square$

### Remark 6.3

To obtain a better estimate of $\beta$, one should construct an initial value layer, since $\beta |_{t=0}\not = 0$.

## Appendix B. Model of early carcinogenesis – constant steady states

Here, we consider the space homogeneous solutions of the two-equation model (6.6)–(6.10) which satisfy the corresponding kinetic system

7.1 $$\begin{aligned} u_t&= \left( \dfrac{auw}{d_b + d + uw} - d_c \right) u, \end{aligned}$$

7.2 $$\begin{aligned} w_t&= - d_g w -\dfrac{d_b}{d_b + d}u^2w + \kappa _0, \end{aligned}$$

where *a*, $d_c$, $d_b$, *d*, $d_g$, $\kappa _0$ are positive constants and we have always assumed that $a> d_c$. The structure of constant steady states of this system is the same as of the original three-equation model and can be characterized by the following lemma (for the proof see Marciniak-Czochra et al. (2013)).

### Lemma 7.1

Let $\Theta = 4d_g \left( \dfrac{d_c}{a-d_c}\right) ^2 d_b (d_b + d)$. If $\kappa _0^2 > \Theta$ , then system (7.1)–(7.2) has two positive steady states $(u_- , w_-)$ and $(u_+ , w_+)$ with

7.3 $$\begin{aligned} u_\pm = \dfrac{d_c}{a-d_c}(d_b + d)\dfrac{1}{w_\pm } \quad \text {and}\quad w_\pm = \dfrac{\kappa _0 \pm \sqrt{\kappa _0^2 - \Theta }}{2d_g}. \end{aligned}$$

### Theorem 7.2

Let $(u_- , w_-)$ and $(u_+ , w_+)$ be positive steady states of system (7.1)–(7.2) given by (7.3). Then $(u_+ , w_+)$ is always unstable. While $(u_- , w_-)$ is stable, except for the case

$$\begin{aligned} \dfrac{d_c}{a}(a-d_c)-d_g > 0, \quad \dfrac{\beta }{2} \le 1 \quad \text {and} \quad \kappa _0^2 \le \dfrac{\beta ^2 \Theta }{4(\beta -1)}, \end{aligned}$$

where

$$\begin{aligned} \beta = \dfrac{\dfrac{d_c}{a}(a-d_c)}{\dfrac{d_c}{a}(a-d_c) -d_g} > 1. \end{aligned}$$

### Proof

Let $(\bar{u}, \bar{w})$ denote a steady state of (7.1)–(7.2). From direct calculations, the Jacobian matrix *J* at $(\bar{u}, \bar{w})$ of the nonlinear mapping defined by the right-hand side of (7.1)–(7.2) is of the form

$$\begin{aligned} J = \left( \begin{array}{cc} \displaystyle \dfrac{d_c}{a}(a-d_c) &{} \dfrac{(a-d_c)^2}{a(d_b + d)}\bar{u}^2 \\ -2 \dfrac{d_b d_c}{a-d_c} &{} -d_g - \dfrac{d_b}{d_b + d} \bar{u}^2 \end{array} \right) . \end{aligned}$$

We know (*cf.* Remark 2.4) that

(i) if
  7.4 $$\begin{aligned} \dfrac{d_c}{a}(a-d_c) -d_g - \dfrac{d_b}{d_b + d} \bar{u}^2 {<} 0 \quad \text {and}\; - \dfrac{d_g d_c}{a}(a-d_c) {+} \dfrac{d_b d_c (a-d_c)}{a(d_b + d)}\bar{u}^2 > 0, \end{aligned}$$
  then all eigenvalues of *J* have negative real parts;
(ii) if
  7.5 $$\begin{aligned} \dfrac{d_c}{a}(a-d_c) -d_g - \dfrac{d_b}{d_b + d} \bar{u}^2 > 0 \quad \text {or}\quad - \dfrac{d_g d_c}{a}(a-d_c) {+} \dfrac{d_b d_c (a-d_c)}{a(d_b {+} d)}\bar{u}^2 < 0, \end{aligned}$$
  then there is an eigenvalue of *J* which has a positive real part.

*Step 1.* First, we show the stability of $(u_+, w_+)$. Using estimates (7.3), the second inequality of (7.5) can be written in the form

7.6 $$\begin{aligned} \dfrac{d_b (d_b + d)}{d_g}\left( \dfrac{a-d_c}{d_c}\right) ^2 < \bar{w}^2. \end{aligned}$$

Note that the left-hand side of (7.6) satisfies

$$\begin{aligned} \dfrac{d_b (d_b + d)}{d_g}\left( \dfrac{a-d_c}{d_c}\right) ^2 = \frac{\Theta }{4 d_g^2}. \end{aligned}$$

and $w_+$ satisfies $\left( \kappa _0 / (2d_g)\right) ^2< w_+^2 < \left( \kappa _0 / d_g \right) ^2$. Therefore, it follows from the assumption $\kappa _0^2 > \Theta$ that

$$\begin{aligned} \frac{\Theta }{4 d_g^2}< \frac{\kappa _0^2}{4 d_g^2} < w_+^2, \end{aligned}$$

which implies that the steady state $(u_+, w_+)$ is unstable.

*Step 2.* Next, we show stability of $(u_- , w_-)$. The second inequality of (7.4) is equivalent to $\Theta / (4d_g^2) > \bar{w}^2$. The latter inequality holds true, since $w_-^2 = \frac{2\kappa _0^2 - 2\kappa _0 \sqrt{\kappa _0^2 - \Theta } - \Theta }{4d_g^2}$ and

$$\begin{aligned} \Theta - \left( 2\kappa _0^2 - 2\kappa _0 \sqrt{\kappa _0^2 - \Theta } - \Theta \right) = 2\sqrt{\kappa _0^2 - \Theta } \left( \kappa _0 - \sqrt{\kappa _0^2 - \Theta } \right) > 0. \end{aligned}$$

Using (7.3) and the relationship $d_b (d_b + d)\left( \frac{d_c}{a-d_c}\right) ^2 = \frac{\Theta }{4d_g}$, the first condition of (7.4) becomes

$$\begin{aligned} w_-^2 \left[ \frac{d_c}{a} (a-d_c) - d_g \right] < \frac{\Theta }{4d_g}. \end{aligned}$$

If $\frac{d_c}{a} (a-d_c) - d_g \le 0$, then the above inequality always holds.

Assume $\frac{d_c}{a} (a-d_c) - d_g > 0$. Note that $w_-^2$ is given by

$$\begin{aligned} w_-^2 = \dfrac{2\kappa _0^2 - 2\kappa _0 \sqrt{\kappa _0^2 - \Theta } - \Theta }{4d_g^2}. \end{aligned}$$

Therefore, it is sufficient to show the following inequality

7.7 $$\begin{aligned} \left[ 2\kappa _0^2 - 2\kappa _0 \sqrt{\kappa _0^2 - \Theta } - \Theta \right] \left[ \frac{d_c}{a} (a-d_c) - d_g \right] < d_g \Theta . \end{aligned}$$

The left-hand side of (7.7) is

$$\begin{aligned}&\left[ 2\kappa _0^2 - 2\kappa _0 \sqrt{\kappa _0^2 - \Theta } - \Theta \right] \left[ \frac{d_c}{a} (a-d_c) - d_g \right] \\&\quad = \dfrac{d_c}{a}(a-d_c)\left[ 2\kappa _0^2 - 2\kappa _0 \sqrt{\kappa _0^2 - \Theta } - \Theta \right] - d_g \left[ 2\kappa _0^2 - 2\kappa _0 \sqrt{\kappa _0^2 - \Theta } \right] + d_g \Theta . \end{aligned}$$

Hence, if

7.8 $$\begin{aligned} \dfrac{d_c}{a}(a-d_c)\left[ 2\kappa _0^2 - 2\kappa _0 \sqrt{\kappa _0^2 - \Theta } - \Theta \right] - d_g \left[ 2\kappa _0^2 - 2\kappa _0 \sqrt{\kappa _0^2 - \Theta } \right] < 0, \end{aligned}$$

then inequality (7.7) holds true. Noting $\frac{d_c}{a}(a-d_c) - d_g > 0$, we obtain, from (7.8), that

7.9 $$\begin{aligned} 2\left[ \kappa _0^2 - \kappa _0 \sqrt{\kappa _0^2 - \Theta } \right] < \dfrac{\dfrac{d_c}{a}(a-d_c)}{\dfrac{d_c}{a}(a-d_c) - d_g } \Theta = \beta \Theta , \end{aligned}$$

what is equivalent to

7.10 $$\begin{aligned} \kappa _0^2 -\dfrac{\beta }{2} \Theta < \kappa _0 \sqrt{\kappa _0^2 - \Theta }. \end{aligned}$$

If $\beta /2 > 1$ and $\Theta < \kappa _0^2 \le (\beta /2)\Theta$, then inequality (7.10) is always satisfied since the right-hand side of (7.10) is positive. The remaining cases are (i) $\beta /2 \le 1$ and (ii) $\beta / 2 > 1$ and $\kappa _0^2 > (\beta / 2) \Theta$. In the cases (i) and (ii), the both-sides of (7.10) are positive. Therefore, we calculate the square of both sides of (7.10) and obtain

7.11 $$\begin{aligned} \frac{\beta ^2}{4 (\beta - 1)} \Theta < \kappa _0^2. \end{aligned}$$

If $\beta > 2$, then $\beta / 2 > \beta ^2 / (4(\beta - 1))$, while $\beta / 2 \le \beta ^2 / (4(\beta - 1))$ if $\beta \le 2$. Therefore, the inequality (7.10) holds in case (ii). In case (i), (7.10) is satisfied under the condition (7.11). $\square$
